# Efficacy and Safety of Immunotherapeutic Strategies for the Treatment of MART-1-Expressing Melanoma: Systematic Review of Clinical Trials

**DOI:** 10.3390/cancers18183016

**Published:** 2026-09-17

**Authors:** Alina Alshevskaya, Sofya Tsarikova, Elena Golikova, Peter Timashev, Sergey Sennikov

**Affiliations:** 1 Federal State Autonomous Educational Institution of Higher Education, I.M. Sechenov First Moscow State Medical University of the Ministry of Health of the Russian Federation (Sechenov University), Moscow 119048, Russia; tsarikova_s_i@staff.sechenov.ru (S.T.); golikova_e_a@staff.sechenov.ru (E.G.); timashev_p_s@staff.sechenov.ru (P.T.); sennikovsv@gmail.com (S.S.); 2 Federal State Budgetary Scientific Institution “Research Institute of Fundamental and Clinical Immunology”, Novosibirsk 630099, Russia

**Keywords:** adoptive cell therapy, autologous cell transfer, dendritic cell vaccines, DNA vaccines, immunotherapy, MART-1, Melan-A, melanoma, peptide vaccines, TCR-T therapy

## Abstract

**Background/Objectives**: Melanoma antigen recognized by T cells 1 (MART-1) is a tumor-associated antigen overexpressed in melanoma cells that serves as a target for different immunotherapeutic strategies. These approaches differ substantially in their efficacy and safety and are associated with various immunologic challenges, including the incidence of autoimmunity. Although multiple clinical trials evaluated anti-MART-1 immunotherapies, the available evidence is highly heterogeneous and requires systematic evaluation. The present study aimed to systematically summarize and descriptively compare the reported efficacy and safety of different MART-1-targeted immunotherapeutic strategies. **Methods**: Clinical trial registries and bibliographic databases were systematically searched to identify clinical trials investigating anti-MART-1 immunotherapies. Data on the objective response rate (ORR), median relapse-free survival (RFS), overall survival (OS), progression-free survival (PFS), time to progression (TTP), and adverse effects were extracted and structured. Potential immunologic challenges underlying the observed outcomes were also discussed. **Results**: Fifty-four clinical trials comprising 1292 treated patients were included, of whom 517 entered their trial with measurable disease and were evaluable for an objective response by RECIST. Higher objective response rates were reported in the autologous cell transfer and TCR-T trials than in the peptide- and DNA-based vaccine trials, whereas the vaccine trials reported fewer immune-related adverse events. These are uncontrolled cross-trial observations. The cell-based regimens were administered after non-myeloablative lymphodepletion and usually with high-dose IL-2, whereas the vaccination protocols were not, so the differences between modalities cannot be attributed to MART-1 targeting alone. No meta-analysis and no formal comparative efficacy analysis were performed. **Conclusions**: Based on the available data, MART-1-targeted immunotherapeutic strategies showed distinct efficacy and safety profiles. Higher objective response rates were reported in some adoptive cell therapy trials, together with a higher frequency of immune-related adverse events; these are uncontrolled cross-trial observations, and the contribution of concomitant lymphodepletion and IL-2 cannot be separated from that of MART-1 targeting. Peptide- and DNA-based vaccine approaches were associated with fewer immune-related adverse events and may warrant further evaluation as components of combined regimens.

## Simple Summary

Immunotherapy has become an important treatment strategy for patients with advanced melanoma. Melanoma antigen recognized by T cells 1 (MART-1) has been extensively investigated as a target for a variety of immunotherapeutic approaches, including DNA- and peptide-based vaccines, adoptive cell therapies, and combination strategies. However, the clinical efficacy and safety of these approaches have not been systematically compared. This systematic review summarizes the available evidence from published clinical trials and describes the reported efficacy and safety of different MART-1-targeted immunotherapies. Higher objective response rates were reported in some adoptive cell therapy studies, whereas vaccine studies generally reported fewer immune-related adverse events. These observations require cautious interpretation because treatment regimens, patient populations, and adverse-event reporting differed across studies. These findings provide an overview of the current clinical evidence and may support the future development of more effective and safer MART-1-targeted immunotherapies for melanoma.

## 1. Introduction

Since melanoma is a heterogeneous disease, melanoma treatment strategies are guided by several factors: disease stage and dissemination, resectability, BRAF V600 mutation status (the only melanoma-associated mutation targetable with inhibitors), the patient’s prior therapy and current condition. The cornerstone of melanoma treatment is surgical resection, when feasible, along with target immunotherapy, represented by immune checkpoint inhibitors targeting PD-1, CTLA-4, and LAG-3, as well as BRAF and MEK inhibitors for tumors bearing BRAF V600 mutation [1].

Novel treatment modalities include cell therapy, personalized mRNA vaccines, and TCR targeted medications. Commercially available products include autologous TIL-based Lifileucel (Amtagvi, USA) [2]; and tebentafusp (Kimmtrak), an ImmTAC, that is, a soluble affinity-enhanced T-cell receptor fused to an anti-CD3 single-chain variable fragment, which is directed at a gp100-derived peptide presented by HLA-A*02:01 and is approved for unresectable or metastatic uveal melanoma; tebentafusp does not target MART-1 [3]. Personalized neoantigen therapy with mRNA vaccine V940/mRNA-4157 is currently in phase III clinical development [4].

If the primary melanoma is thick, ulcerated or has spread to regional lymph nodes, systemic therapy following surgical removal is recommended. If complete resection is impossible or tumor cells disseminated to distant regions, systemic immunotherapy becomes the primary treatment option [5]. However, among systemic treatment methods, cell-based options are not considered first-line therapy [2].

Although current melanoma treatments have improved clinical outcomes, the identification of reliable melanoma-associated antigens and development of targeted immunotherapies could further ensure more precise and effective tumor elimination [6]. Melanoma antigen recognized by T cells 1 (MART-1) is a tumor-associated antigen, overexpressed in melanoma cells and presented in up to 80–95% of melanoma cell lines [1,7]. MART-1 is a lineage-restricted melanocyte differentiation antigen of 118 amino acids [8]. Biologically, MART-1 is a type III signal-anchor protein localized mainly in the trans-Golgi network. MART-1 is involved in melanogenesis, where it forms a complex with PMEL17/gp100 [9], regulating its expression, stability, and processing [10,11].

MART-1 is presented in the context of the HLA-A2 allele, shared by many human populations across the world, thereby ensuring the broad applicability of MART-1-targeted immunotherapies [12]. However, computer algorithms predict that MART-1 immunogenic epitopes bind to other HLA supertypes, a finding that may have implications for future clinical trials by enabling the inclusion of patients beyond HLA-A2-positive individuals [13].

A naïve MART-1-specific CD8 T-cell repertoire is present in healthy individuals, but may develop a cytotoxic phenotype upon encountering an antigen, resulting in autoimmunity or an antitumor response to MART-1-expressing tumors [14]. This observation is potentially attributable to the misinitiation of MART-1 expression in the human medullary thymic epithelial cells, which produces a truncated mRNA product; accordingly, the corresponding peptide lacks the immunodominant 26–35 epitope. Consequently, a highly diverse MART-1 26–35-specific naive T-cell repertoire that develops naturally evades negative selection and persists in the peripheral blood [15]. The pre-existing baseline level of MART-1-cytotoxic lymphocytes holds practical significance, as it enables the evaluation of pre-existing immunity prior to immunotherapy and facilitates longitudinal monitoring of cellular immune responses to treatment [16]. Moreover, the presence of MART-1-specific TCRs and their population composition may potentially serve as an important prognostic marker and even aid in diagnosing melanoma patients through predictive models [13].

The natural MART-1 immunogenic epitopes are a nonameric peptide spanning residues 27–35 (MART-1 27–35: AAGIGILTV) and a decapeptide (MART-1 26–35: EAAGIGILTV) that includes one additional residue at the NH2 terminus [17]. The extra amino acid causes the decameric epitope to acquire a bulging conformation in the MHC groove, distinct from the extended conformation of the nonapeptide [18]. Unmodified decameric and nonameric MART-1-derived epitopes are generally weak agonists, as the absence of an anchor residue at the second position results in suboptimal HLA binding, leading to instability of peptide–MHC complexes and inferior immune recognition [19]. Accordingly, numerous attempts were made to modify the initial epitope structure. Leucine or alanine substitutions improve the stability of the peptide–MHC complex, thereby improving recognition by a large number of T-cell populations, complete activation, and potent cytotoxic activity. Further considerations on the optimal epitope structure and attempts to design novel superagonist epitopes continue to enhance clinical outcomes of immunotherapy [19,20].

The widespread expression of MART-1 across tumor lines, its presentation via common HLA-A2 alleles, and its ability to induce a cytotoxic T-cell repertoire with traceable immune responses make this antigen an attractive immunotherapeutic target, facilitating the development of diverse immunotherapeutic approaches.

MART-1-directed immunotherapies fall into two main categories: adoptive cell therapies (ACTs) and non-ACT approaches. In non-ACT therapies, the anti-MART-1 immune response is induced via vaccines comprising peptides, DNA molecules, or viral vectors. In contrast, ACT therapies utilize living cells as therapeutic agents, such as antigen-presenting cells (dendritic cells (DCs) or monocyte-derived dendritic cells (moDCs)), autologous or allogeneic genetically modified T-lymphocytes (as seen in TCR-T therapy), and antigen-reactive peripheral blood mononuclear cells or tumor-infiltrating lymphocytes, which naturally develop in the body in response to tumor cells [21]. These approaches have shown varying capacities to induce clinically significant effects by generating MART-1-specific clones of lymphocytes, improving patient responses, and prolonging survival. However, these therapies also face serious challenges, including the risk of inducing autoimmune reactions due to the destruction of MART-1-bearing healthy cells, potentially leading to the development of uveitis, hypopigmentation and hearing abnormalities, as well as cytokine release syndrome caused by hyperactivation of immune cells. Thus, there is a growing need to assess the therapeutic potential and safety profile of such immunotherapeutic strategies.

Numerous clinical trials have evaluated MART-1-directed immunotherapies, but the available evidence is fragmented and heterogeneous in terms of treatment protocols, patient populations, and outcome reporting. This systematic review aimed to capture the breadth of the available clinical evidence and provide a structured descriptive comparison of the reported efficacy and safety outcomes across MART-1-directed strategies. By bringing these diverse findings together, we sought to identify patterns warranting further investigation and gaps in outcome reporting that limit comparability and should be addressed in future studies.

## 2. Materials and Methods

### 2.1. Data Source and Search Strategy

This study employed data obtained from four sources: the bibliographic databases Scopus and PubMed/MEDLINE, and the clinical trial registries ClinicalTrials.gov and the EU Clinical Trials Register. Scopus served as the primary bibliographic source; PubMed/MEDLINE was searched with the same title-based term set as Scopus to retrieve records not indexed in Scopus; the two registries were searched to identify trials irrespective of publication status; and the reference lists of the retrieved full texts were additionally screened by citation searching. The last search in all four sources was performed on 15 January 2026. The exact search string used in each source and the number of records retrieved are reported in Table 1. In the Scopus database, the search query “TITLE ((trial OR study OR clinical OR treatment) AND (MART-1 OR MART1 OR (melanoma antigen recognized)))” yielded a list of 317 articles. The same term set was applied in PubMed/MEDLINE as the query (“MART-1”[Title] OR “MART1”[Title] OR “melanoma antigen recognized”[Title]) AND (trial[Title] OR study[Title] OR clinical[Title] OR treatment[Title]), which yielded 25 records. The articles then were manually filtered to select those reporting outcomes of clinical trials evaluating MART-1-targeted immunotherapies.

Clinical trials were extracted from the registries using the query “MART-1” OR “Melan-A” for the EU Clinical Trials Register and the query (“Melan-A” OR “MART-1” OR “MART1” OR “melanoma antigen recognized”) AND (“melanoma” OR “skin cancer”) for ClinicalTrials.gov.

The lists retrieved from trial registers and bibliographic databases were combined and thoroughly reviewed. Trial register entries with no available results were excluded unless the missing information could be retrieved by searching the literature databases.

During the preparation of this work the authors used GPT-5.5 in order to improve language and graphical abstract. After using this tool/service, the authors reviewed and edited the content as needed and take full responsibility for the content of the publication abd study methodology.

### 2.2. Inclusion and Exclusion Criteria

According to inclusion criteria, the analysis encompassed clinical trials of immunotherapies targeting MART-1-expressing melanoma with any number of patients including single group or parallel, randomized or non-randomized, interventional or observational trials, involving retrospective analyses, and case reports describing outcomes following MART-1-targeted immunotherapies. Irrelevant trials and trials with no available results were excluded from the analysis. The process of clinical trial systematic search and screening was performed by two reviewers independently (S.T. and A.A.). Any disagreements on trial inclusion and exclusion were resolved by a third reviewer (E.G.).

### 2.3. Data Extraction

Data extraction was carried out by two researchers independently. Key trial characteristics, such as the name or identifier of the study, start year, number of treated patients, and treatment modality, were extracted from each trial; the outcomes of interest involved the objective response rate (ORR), data on median RFS, PFS, TTP, and OS, and incidence and nature of adverse events. The key characteristics of the included trials were summarized in a table for comprehensive overview.

### 2.4. Key Clinical Outcomes

In clinical trials reporting the number of objective responses to therapy according to RECIST (Response Evaluation Criteria in Solid Tumors), the following outcomes were calculated: objective response rate (ORR), defined as the sum of partial and complete responses to therapy divided by the number of patients evaluable for response; if provided, disease stabilization (SD), progression of disease (PD), and mixed response (MR) rates were calculated similarly. Calculations considered only patients evaluable for response by RECIST criteria who entered the trial with measurable disease. In clinical trials reporting RFS, PFS, TTP, or OS, the median value was extracted in days. For clinical trials not reporting the median value but providing the individual patient data, the Kaplan–Meier method was performed to estimate the median. Throughout the manuscript, the number of treated patients and the number of patients evaluable for response are reported separately: the term evaluable refers exclusively to patients who entered a trial with measurable disease assessable by RECIST, and only these patients contribute to the denominator of an objective response rate, whereas trials that reported no response data contribute to the treated count only. A narrative, descriptive synthesis was planned. No meta-analysis and no formal comparative efficacy analysis were performed, because the number of trials per modality is small and the trials differ substantially in design, phase, patient population, concomitant treatment and outcome reporting, a situation in which pooling is explicitly discouraged (Cochrane Handbook for Systematic Reviews of Interventions, Chapter 10, Section 10.10.3). The review was reported in accordance with the PRISMA 2020 statement [22].

Adverse effects were classified according to CTCAE (Common Terminology Criteria for Adverse Events). For each group, the total number of adverse effects was calculated. Trials that provided only a descriptive summary without specifying the number of AEs or affected patients were excluded from the calculations. In studies where only the number of patients with a specific adverse event was reported, this was treated as one event per affected patient for the purpose of AE/patient calculation. Based on literature findings, uveitis, vitiligo (skin depigmentation), hearing impairment, and cytokine release syndrome were classified by the authors as immune-related adverse events [23,24,25]. Within this group, uveitis, vitiligo and hearing impairment are considered possible on-target, off-tumor effects of reactivity against MART-1-expressing healthy cells in the skin, the uveal tract and the inner ear, whereas cytokine release syndrome reflects systemic immune activation rather than direct targeting of MART-1-expressing tissue. The two mechanisms are counted together as immune-related events but are discussed separately. In each case, the adverse event rate or frequency of adverse events belonging to the certain category was expressed as the number of adverse events per one patient (AEs/patient). The two adverse event metrics were therefore calculated on different denominators: the overall AE/patient rate and the rates for individual AE categories include only those trials that reported the exact number of all adverse events, whereas the frequency of immune-related adverse events additionally includes trials that reported such events descriptively without providing a numeric total, so that the denominator of each rate is the number of patients in the trials contributing to that particular rate and is stated alongside it.

### 2.5. Risk of Bias

No formal risk of bias assessment of the included studies was performed. The included set consists almost entirely of single-arm, early-phase, non-randomized studies; a number of records were available only as trial registry entries without a corresponding full-text publication, and two records are single-patient case reports. The information required by standard risk-of-bias instruments, such as the method of randomization, allocation concealment, blinding, the presence of a comparator arm and pre-specified outcomes, is not reported for most of these records. Instead, the design, phase, sample size and data source of every included record are given in Supplementary Table S1, and the recurring methodological limitations are discussed qualitatively in the Discussion.

## 3. Results

### 3.1. Search Results

Publication extraction with the search strategy described in Section 2.1 was performed on 15 January 2026 and yielded 317 potentially relevant articles from Scopus. A parallel search in PubMed/MEDLINE with the same title-based term set retrieved 25 records, 24 of which had already been identified in Scopus; the single additional record did not describe a clinical trial of MART-1-targeted immunotherapy and was excluded at title screening. Seventy-three records were removed before screening: 24 duplicates identified in both databases, 48 non-article publications, such as conference papers, erratums, and notes, and 1 retracted article, leaving 269 articles for subsequent screening. Articles were manually screened to extract those reporting results of clinical trials evaluating MART-1-immunotherapies. Twenty-nine articles were selected for full-text screening. The full text of one of them, the pilot trial of a MART-1 peptide vaccine reported by Block et al. [26], could not be obtained: neither the publisher nor the authors provided access to the complete report, and the abstract alone did not contain the outcome data required for extraction. This report was therefore recorded as not retrieved and was not included in the analysis. Citation searching of the reference lists of the remaining 28 articles identified 22 further eligible publications, so that 50 articles were retained for systematic analysis.

Database search in ClinicalTrials.gov and the EU Clinical Trials Register with the search query and keywords yielded 93 clinical trials. The decision on including the entries with absent results in the further analysis was based on whether attempts to restore data by a literature search were successful; if the entry had a corresponding article, it was added to the final list.

**Figure 1:**
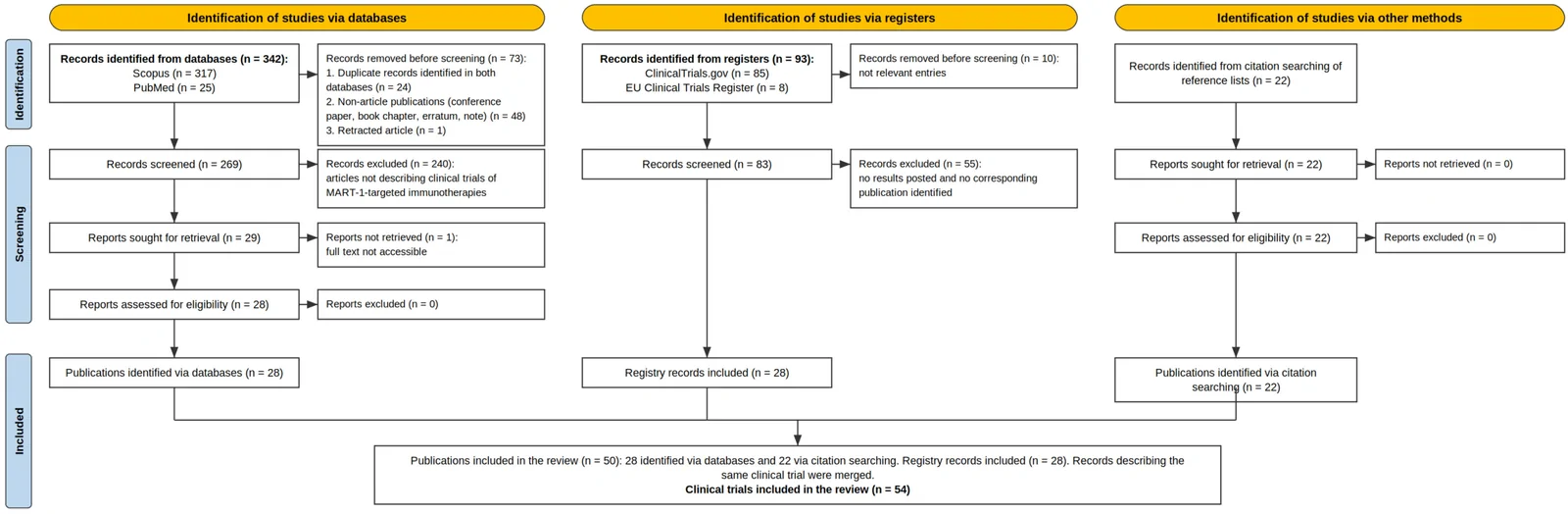
The PRISMA flow diagram demonstrating study identification and selection process.

Both lists obtained by a literature and register search were cross-referenced, and those entries related to one clinical trial were merged, which yielded a set of 54 unique clinical trials. The PRISMA flow diagram depicting the whole process of trials’ identification and selection, including the reasons for the exclusion, is presented in Figure 1. The search queries for each database and number of obtained results are presented in Table 1.

### 3.2. Overall Trial Characteristics

**Figure 2:**
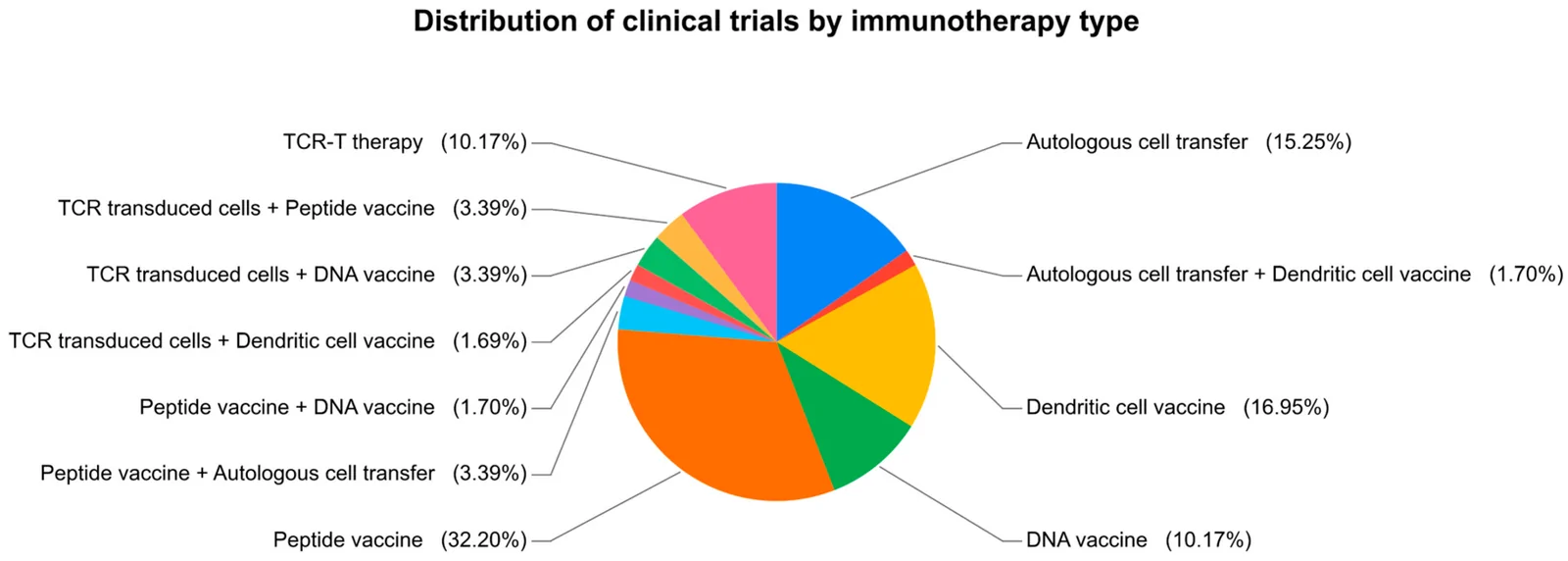
The pie chart showing distribution of the immunotherapy types among the reviewed studies.

**Figure 3:**
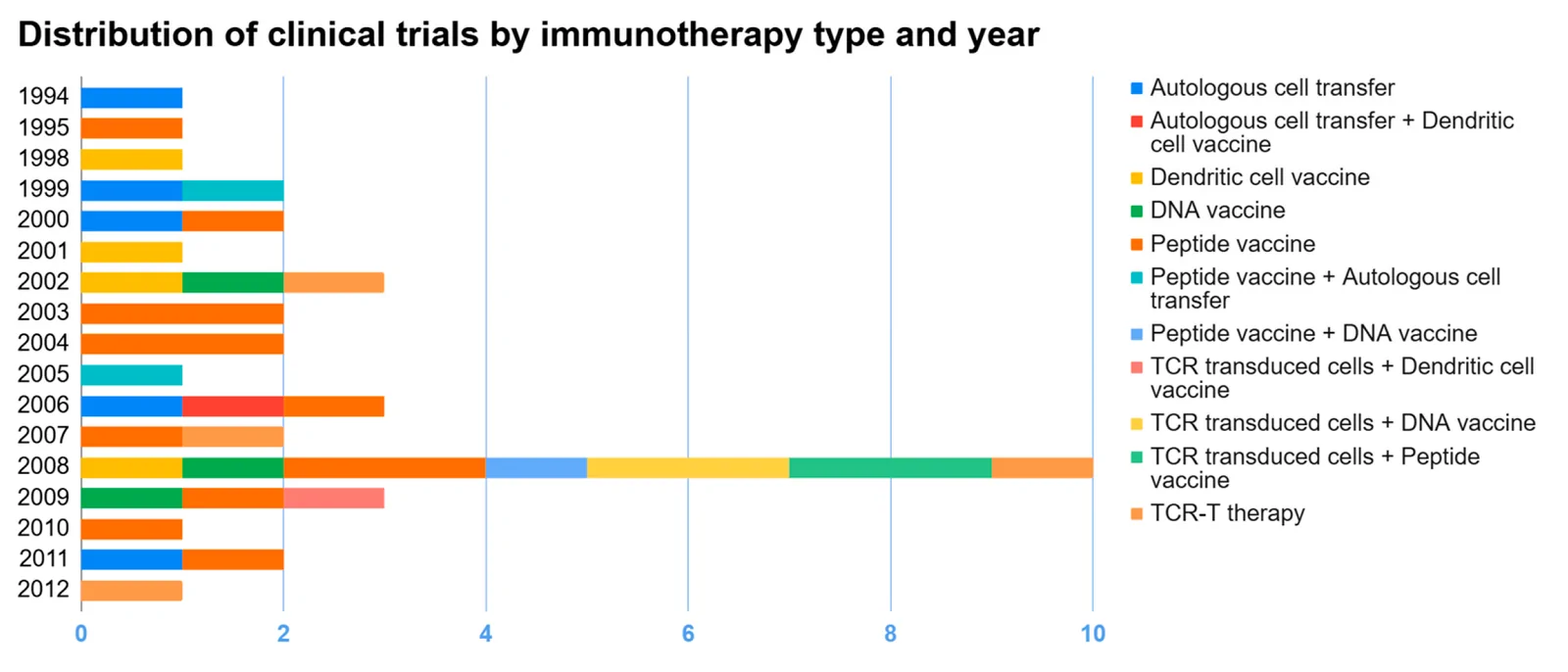
Bar chart illustrating the year distribution of clinical trials with evaluated therapy formulations specified.

The database search yielded 54 CTs. The search was not restricted by publication date or by trial start year, and the included set comprises trials initiated from 1994 onwards. Among the identified trials investigating immunotherapy of MART-1-expressing melanomas, the evaluated treatments were grouped as follows: peptide vaccines (*n* = 19), autologous cell infusion (*n* = 9), TCR-T therapy (*n* = 6), DNA vaccines (*n* = 6), dendritic cell vaccines (*n* = 10), and combination approaches. The latter group comprised several subcategories: (1) ACT combinations, represented by TCR-T therapy with DC vaccine (*n* = 1) and autologous cell transfer with DC vaccine (*n* = 1); (2) combinations of ACT and non-ACT approaches, including TCR-T therapy with peptide vaccines (*n* = 2) or DNA vaccines (*n* = 2), and autologous cell infusion with peptide vaccines (*n* = 2); (3) non-ACT combination of DNA vaccine with peptide vaccine (*n* = 1). As some trials evaluated several types of immunotherapy across separate cohorts, such trials can be represented more than once in different categories. The 54 trials comprised 1292 treated patients with skin, mucosal, and ocular melanomas. A trial that evaluated several modalities in separate cohorts is counted once in the total number of trials, while each of its cohorts contributes separately to the corresponding modality group; the modality figures are therefore cohort-level and sum to the overall total. Of the 1292 patients, 517 entered their trial with measurable disease and were evaluable for an objective response by RECIST. The comprehensive table of clinical trials’ key characteristics and outcomes, including data on the response to therapy, is presented in the Supplementary Materials (Table S1). The year distribution and relative proportions of clinical trials investigating different MART-1-targeted immunotherapies are shown in Figure 2 and Figure 3.

**Figure 4:**
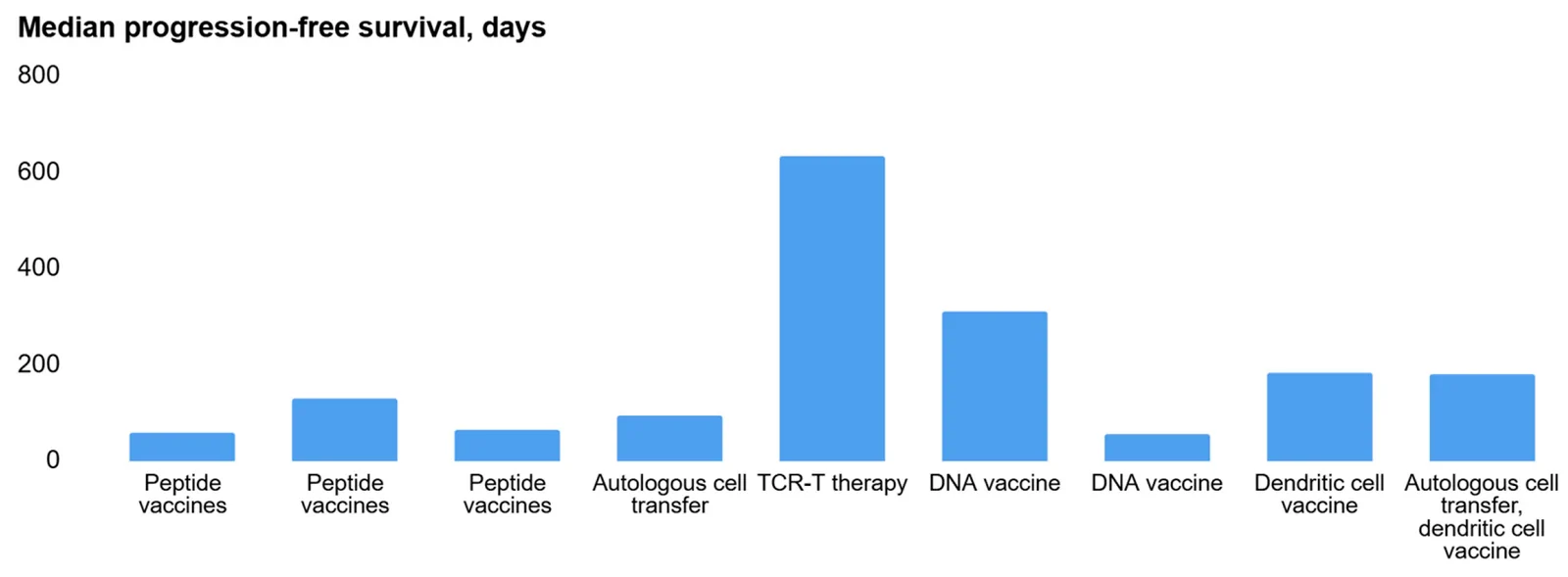

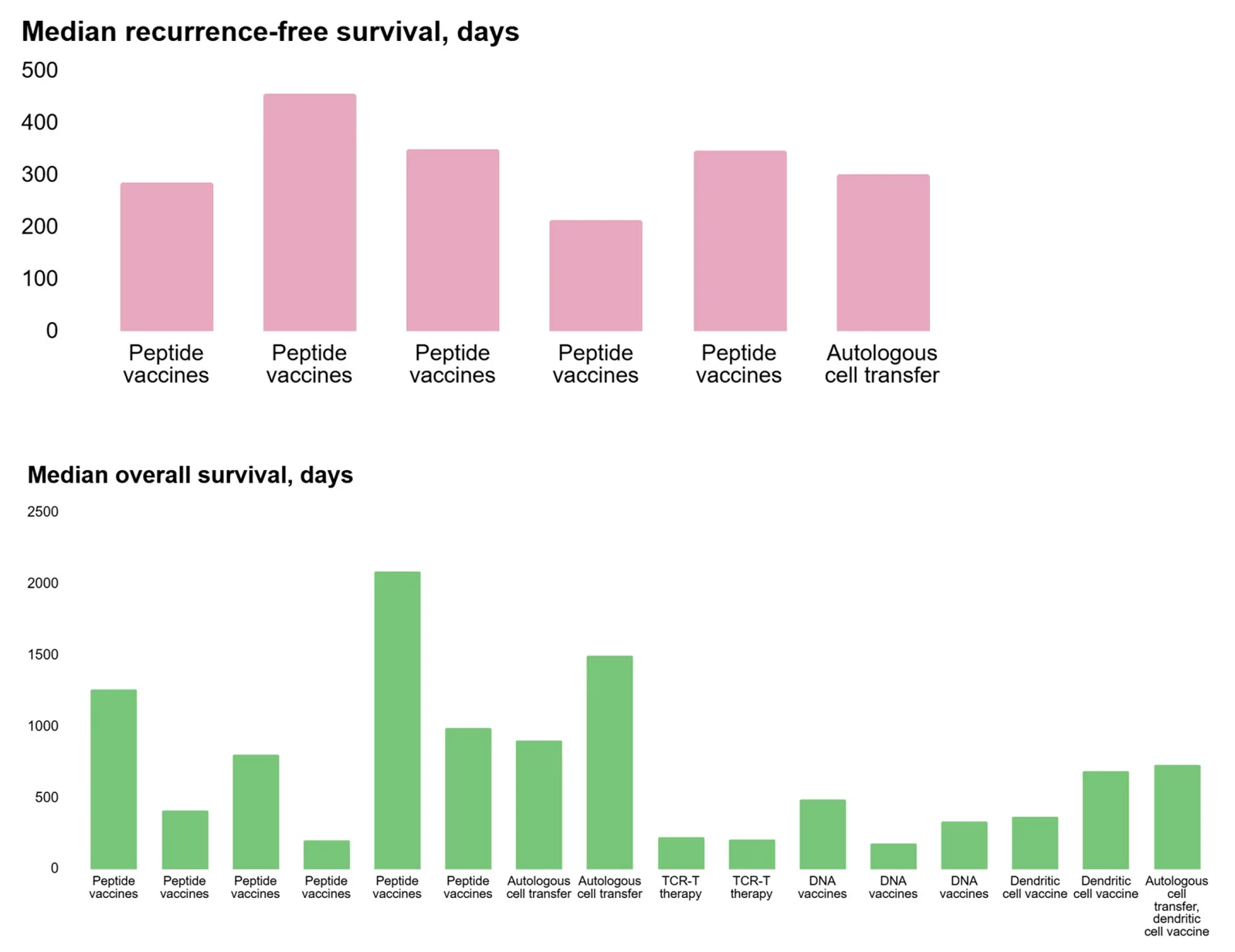
Histograms representing the median duration of progression-free survival, relapse-free survival, and overall survival in days. Each bar represents a clinical trial reporting a given parameter, with therapy formulation specified.

Among outcomes of interest, 39 (72%) trials reported the objective response to therapy; 16 (30%) clinical trials reported median RFS, PFS, TTP, or OS. Clinical trials that achieved an objective response to therapy are summarized in Table 2, and median survival measurements across clinical trials are depicted in the bar charts of Figure 4.

**Figure 5:**
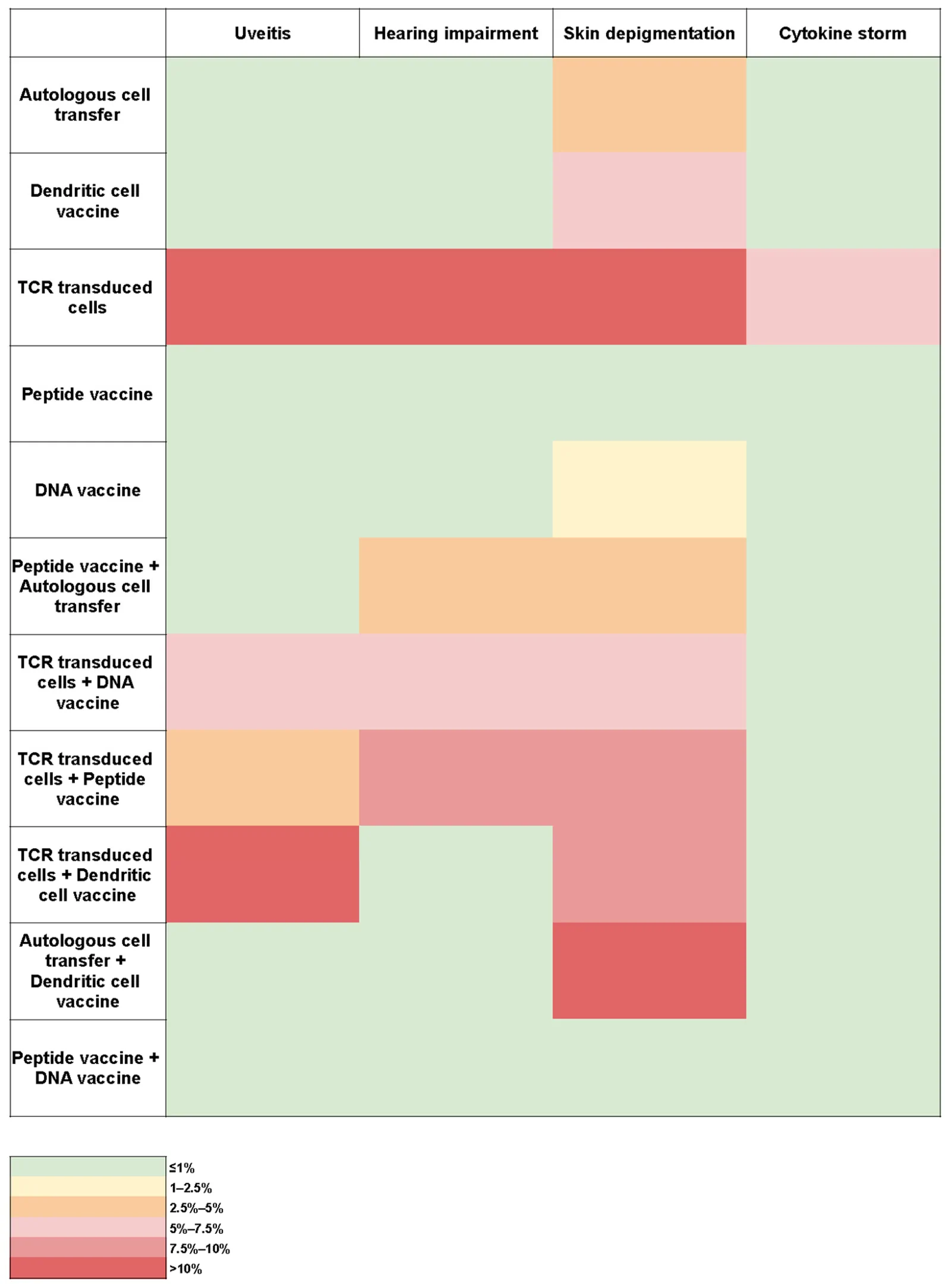
The bar chart represents the frequency of certain immune-related adverse effects for each type of immunotherapy.

**Figure 6:**
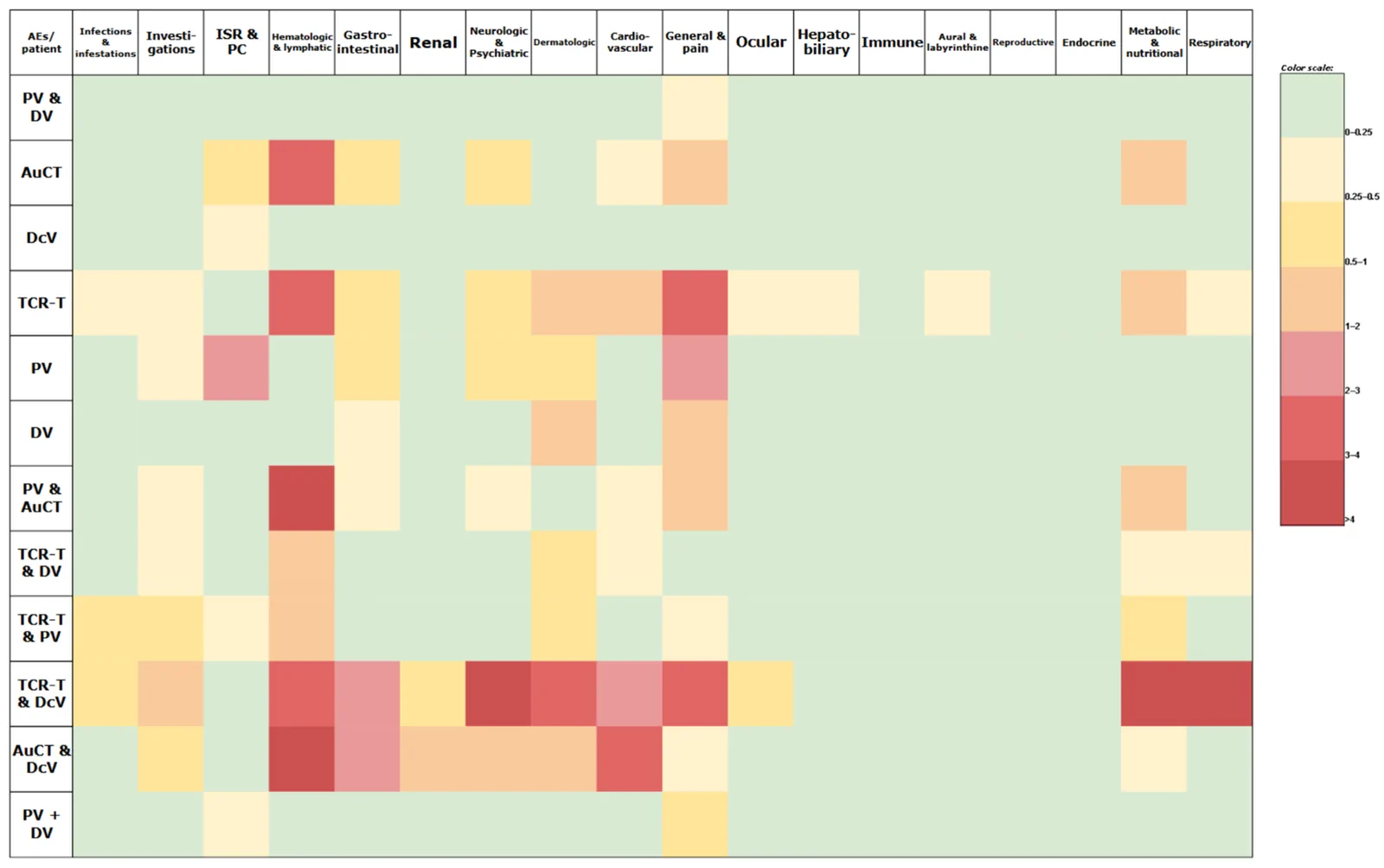
Heatmap depicting the frequencies of certain adverse effect categories across different immunotherapy types. Cell color ranges from green to red, indicating the lowest and the highest frequencies of the certain adverse effect category, respectively, as indicated by the color scale. AuCT—autologous cell transfer, PV—peptide vaccines, DV—DNA vaccines, DcV—dendritic cell vaccines, ISR and PC—injection site reactions and procedural complications.

Forty-nine CTs (91%) provided information about adverse events. Uveitis, hearing impairment, skin depigmentation, and cytokine release syndrome were classified by the authors as immune-related. It is important to distinguish between the on-target/off-tumor effects of MART-1 targeting (uveitis, vitiligo, hearing impairment) and cytokine release syndrome, which reflects systemic immune activation rather than direct damage to MART-1-expressing normal cells. Both categories were included among immune-related adverse events, but their mechanistic differences are acknowledged. The distribution of these adverse effects for different immunotherapies is presented in Figure 5. Figure 6 contains a heatmap depicting the frequencies of each adverse effect category across immunotherapy formulations. In Figure 5 and Figure 6, cytokine release syndrome is labeled as cytokine storm.

Other endpoints reported in clinical trials but not included in the present analysis were the potency and durability of the anti-MART-1 immune response.

Some clinical trials did not rely on strictly anti-MART-1-oriented immune cells to induce immunity; instead, they employed lymphocytes isolated for melanoma cell reactivity rather than affinity for specific antigens, or infused dendritic cells loaded with whole tumor oncolysate, permitting a broad, non-specific immune response against multiple antigens expressed by melanoma. The antigen specificity either of the infused therapeutic cells or the therapy-induced immune response was subsequently characterized in these trials. The outcomes of such trials were also considered in the subgroup analysis.

### 3.3. DNA Vaccines

Six clinical trials evaluated immunization with DNA vaccines and included a total of 97 treated patients. Objective responses to therapy were reported in only three trials (Adamina et al. [NCT00116597] [31], Rosenberg et al. [32], Geukes Foppen et al. [48]), which included 60 patients in total. Forty-nine patients were evaluable for response by RECIST; of these, 6 patients (12.24%) achieved objective responses, 8 (16.33%) had stable disease, and the remaining 35 (71.43%) experienced progressive disease. The trial NCT00706992, Triozzi et al. [49], and Weber et al. [50] reported only immune response and toxicity. The exact number of AEs was reported in five trials comprising 61 patients, totaling 219 cases (3.59 AEs/patient). Rosenberg et al. did not provide numeric data, describing mild hepatic transaminase elevations, injection site reactions, and transient AEs associated with administration of high-dose IL-2. One case of vitiligo was observed; thus, the frequency of immune-related AEs was 0.01 per patient, calculated on the 97 patients of the five trials that provided countable adverse event data. Among the AE categories, dermatologic AEs (1.16 AEs/patient) and general AEs including muscle pain (1.15 AEs/patient) had the highest frequencies. DNA vaccines differed with the type of vector, type of MART-1 antigen, and based on whether additional melanoma-associated antigens were included.

#### 3.3.1. Viral Vector-Based Vaccines

Three trials (Adamina et al. [NCT00116597] [31], Rosenberg et al. [32], and NCT00706992) assessed viral vector-based vaccines in a total of 57 patients. One trial (NCT00706992) did not report an objective response to therapy; among the 40 patients evaluable for a response in the other two trials, 6 developed an objective response (15.0%); NCT00706992 contributes to the treated count but not to the response denominator. Only Adamina et al. reported median survival data (PFS 310 days, OS 488.5 days). Adverse events were estimated at 40 cases (1.9 AE/patient), with one immune-related AE (0.02 AEs/patient) reported by Adamina et al.

#### 3.3.2. Plasmid Vector-Based Vaccines

Three trials (Triozzi et al. [49], Weber et al. [50], and Geukes Foppen et al. [48]) assessed plasmid vector-based vaccines in a total of 40 patients. The overall objective response rate for plasmid-based vaccines could not be estimated, as Triozzi et al. and Weber et al. did not report an objective response to therapy, while the trial by Geukes Foppen et al. achieved no objective response. Only Geukes Foppen et al. reported the median PFS (54.8 days) and median OS (179.6 days), while Weber et al. reported the median TTP (61.5 days) and median OS (334.8 days). Adverse events were estimated at 179 cases (4.48 AE/patient).

#### 3.3.3. MART-1 Epitope Variants

DNA vaccines encoded different MART-1 epitopes, including the nonameric MART-1 27–35 epitope, the decameric MART-1 26–35 epitope with leucine substitution (MART-1 26–35 (A27L)) and the long MART-1 31–70 peptide.

Geukes Foppen et al. [48] immunized nine patients with a DNA vaccine encoding the MART-1 26–35 (A27L) epitope but achieved no objective response. No immune-related adverse effects were observed. Adverse events were estimated at 135 cases (15 AEs/patient).

Adamina et al. (NCT00116597) [31] immunized 15 patients with a DNA vaccine encoding the MART-1 27–35 epitope. Among four patients evaluable for a response by RECIST, one experienced an objective response (25%); the trial also reported a median overall survival of 488.5 days. Adverse events were estimated at 38 cases (2.53 AEs/patient). One case of grade I vitiligo was observed (0.067 AEs/patient).

Jeffrey Weber et al. [50] immunized 19 patients with a DNA vaccine encoding two MART-1 epitopes: MART-1 26–35 (A27L) epitope and MART-1 31–70 peptide. Authors reported an onset of the immune response; however, no clinical response was seen. Adverse events were estimated at 42 cases (2.21 AEs/patient).

Other trials did not specify the MART-1 epitope.

According to the available evidence, DNA vaccines encoding the MART-1 26–35 (A27L) epitope were associated with the highest frequency of adverse events (15 AEs/patient); however, this observation is based on a single small clinical trial and should be interpreted cautiously.

#### 3.3.4. Additional Melanoma-Associated Epitopes

Different DNA vaccines also comprised melanoma-associated epitopes, including gp100 and tyrosinase.

Adamina et al. [31] (NCT00116597) immunized 15 patients with the recombinant vaccinia virus encoding fused melanoma-associated antigens gp100, tyrosinase, and MART-1. Among four patients evaluable for a response by RECIST, the trial achieved one objective response (25%). The trial also reported a median PFS of 310 days and a median OS of 488.5 days. Adverse events were estimated at 38 cases (2.53 AEs/patient). One case of grade I vitiligo was observed (0.067 AEs/patient).

Weber et al. [50] immunized 19 patients with a DNA plasmid encoding tyrosinase and MART-1. This study reported a median TTP of 61.5 days, and median OS of 334.8 days. Adverse events were estimated at 42 cases (2.21 AEs/patient). Clinical response was not reported.

### 3.4. Autologous Cell Transfer

Nine trials evaluated autologous cell therapies including MART-1-reactive populations in a total of 198 patients.

The clinical trials by Dudley et al. [35], Dréno et al. [51] and Labarrière et al. [52] (representing the same study) employed the infusion of tumor-reactive cells with a broad range of antigen specificities. Benlalam et al. performed a retrospective study of antigen specificity of the infused cells on a cohort of patients evaluated by Dréno/Labarrière et al., establishing a positive correlation between the presence of MART-1-reactive T cells, and enhanced relapse-free survival in HLA-A2 patients [53].

Eight trials reported the number of objective responses to therapy. In these studies, 154 patients were evaluable for a response by RECIST; 40 patients developed objective responses (26.0%), 11 had stable disease (7.14%), 8 had mixed responses (5.19%), and 95 had progressive disease (61.69%).

Among these trials, Saberian et al. (NCT00338377) [37] reported a median OS (1496.5 days) and median PFS (94.9 days); Dréno/Labarrière et al. also achieved a median RFS of 301.36 days and OS of 898.89 days. Vignard et al. [34] did not provide data on AEs, while Yee et al. [54] reported that 9 of 10 patients experienced myalgias, fever, and fatigue, while one patient developed a targetoid erythematous skin rash. Among remaining trials, 170 patients experienced 1573 AEs (9.25 AE/patient). Seven autoimmune-related adverse effects occurred: one case of uveitis (0.5%) and six cases of vitiligo (3.2%), calculated on the 188 patients of the trials that reported adverse events. The most frequent AE groups were blood and lymphatic system disorders (3.67 AE/patient), general AEs including muscle pain (1.38 AE/patient), metabolism and nutrition disorders (1.21 AE/patient), neurologic/psychiatric AEs (0.85 AE/patient), and gastrointestinal AEs (0.756 AE/patient).

Autologous cell transfer therapies primarily varied according to whether the infused cells were derived from tumor-infiltrating lymphocytes (TILs) or from peripheral blood mononuclear cells (PBMCs). The largest included trial, NCT00001832 (*n* = 89; 23 objective responses, 25.8%), administered TIL- or PBL-derived cells without specifying their source and is therefore not assigned to either subgroup; the TIL subgroup (67 patients), the PBMC subgroup (42 patients) and this trial together account for the 198 patients of this group.

#### 3.4.1. TIL-Derived MART-1-Reactive Cytotoxic Cells

Three trials (Saberian et al. (NCT00338377) [37], Dudley et al. [35], and Dréno/Labarrière et al. [51,52]) used tumor-infiltrating lymphocytes (TILs) in a total of 67 patients. Only one trial by Saberian et al. employed a MART-1-specific T-cell population; Dudley et al. and Dréno/Labarrière et al. administered TIL-derived T-cell clones exhibiting varying specificity for melanoma antigens.

In trials by Saberian et al. and Dudley et al., nine patients in total developed an objective response (39.1%). Saberian et al. (NCT00338377) also reported a median PFS (94.9 days) and a median OS (1496.5 days). The trial by Dréno/Labarrière et al. did not report an objective response, but achieved a median RFS of 301.36 days and median OS of 898.89 days. Dudley et al. reported only five cases of immune-related AEs, corresponding to 0.07 events per patient across the 67 patients of this subgroup, but did not provide total AE data; other two trials reported 556 AEs (10.3 AEs/patient).

#### 3.4.2. PBMC-Derived MART-1-Reactive Cytotoxic Cells

Five trials (NCT01495572, Khammari et al. [NCT00720031] [33], Vignard et al. [34], Yee et al. [54], Meidenbauer et al. [36]) employed MART-1-specific cytotoxic T-lymphocyte clones, generated from a pool of peripheral blood mononuclear cells (PBMCs) in a total of 42 patients. NCT01495572 administered various CD8+ T-lymphocyte populations targeting MART-1, while Khammari et al. (NCT00720031) employed a protocol with isolation of one or two MART-1-specific clones. Eight patients developed an objective response (19%). Among trials that provided numeric AE data, the total number of adverse events was 93 cases (3.44 AE/patient).

Across the trials of this group, higher objective response rates and higher adverse event rates were reported for TIL-derived than for PBMC-derived MART-1-reactive cells. With three and five small trials respectively and no common comparator, this is a descriptive observation and does not establish a difference between the two cell sources.

### 3.5. Dendritic Cell Vaccine

Ten studies investigating dendritic cell (DC) vaccines comprised a total of 112 patients. Three trials (Tuettenberg et al. [55], Terheyden et al. [56], and Tsao et al. [25]) provided no response data assessable by RECIST. The remaining seven trials included 62 patients evaluable for a response by RECIST; among these, 7 patients achieved an objective response (11.3%), 9 patients achieved stable disease (14.52%), and 1 patient had a mixed response (1.61%). Eight trials provided countable adverse event data for 109 patients, totaling 63 cases (0.58 AE/patient). Among the AE categories, injection site reactions and procedural complications (0.4 AE/patient) and dermatologic AEs (0.144 AEs/patient) had the highest frequencies. Eight cases of vitiligo were reported among these 109 patients (7.3%).

DC vaccines consisted of dendritic cells that were either virally transduced or pulsed with tumor lysate and immunogenic peptides.

#### 3.5.1. Oncolysates

Three trials (Palucka et al. [46], Terheyden et al. [56], and Andersen et al. [57]) employed infusion of DCs pulsed with melanoma oncolysates, alone or combined with melanoma peptides, in a total of 23 patients. Palucka et al. demonstrated the induction of functional anti-MART-1 T cells in both HLA-A*02:01 negative and positive patients after vaccination with DCs loaded with killed allogeneic Colo829 melanoma. In a case report, Terheyden et al. described the course of immune response during disease progression in a patient who achieved disease stabilization after administration of a vaccine consisting of dendritic cells pulsed with melanoma-associated peptides (MAGE-3 271–279, gp100 209–217, and MART-1 26–35) and dendritic cells loaded with whole tumor cell lysates. The patient described by Terheyden et al. had no measurable disease assessable by RECIST and therefore contributes to the patient count but not to the response denominator. In the trial by Palucka et al., 2 of 20 evaluable patients achieved an objective response (10.0%), which corresponds to 2 of the 22 patients evaluable for response in this subgroup (9.1%); the median overall survival was 684 days. Andersen et al. vaccinated two patients with dendritic cells pulsed with melanoma peptides alone or combined with autologous oncolysates, reported no objective response, and did not provide countable adverse event data. Palucka et al. also observed one case of vitiligo; thus, the frequency of immune-related AEs was 0.043 per patient.

#### 3.5.2. Peptides

Four studies (Panelli et al. [43], Tuettenberg et al. [55], Butterfield et al. [44], and Ribas et al. [45]) employed infusion of DCs pulsed with peptides in a total of 47 patients. In three trials comprising 21 patients evaluable for response by RECIST, three patients developed an objective response (14.3%); the fourth trial, by Tuettenberg et al., provided no response data assessable by RECIST. Adverse events were estimated at 58 cases (1.23 AE/patient). Three cases of vitiligo (0.06 AE/patient) were observed.

#### 3.5.3. Viral Transduction

Two studies (Tsao et al. [25] and Butterfield et al. [58]) described treatment with adenovirally transduced dendritic cells in a total of 30 patients. Of 12 patients participating in the trial, Tsao et al. presented a report on three patients who developed hypopigmentation after receiving an infusion of adenovirus-mediated gp100/MART-1-transduced dendritic cells. Tsao et al. did not report an objective response to therapy; no objective responses were achieved in trial by Butterfield et al. Adverse events were estimated at 4 cases among the 30 patients of these two trials (0.13 AE/patient). All four recorded events were cases of skin depigmentation (0.13 AE/patient).

#### 3.5.4. RNA Transfection

One study by Dannull et al. (NCT00672542) [47] reported treatment with RNA-transfected DCs derived from iP siRNA-transfected monocytes in 12 patients. The treatment was well-tolerated, and no dose-limiting toxicities or adverse events were observed. Two patients were evaluable for a response by RECIST and both achieved an objective response. This rate rests on the two patients of this trial who had measurable disease and is not comparable with the rates reported by the larger trials.

### 3.6. TCR-T Therapy

Six clinical trials evaluated TCR-T therapy, and included a total of 81 treated patients. An objective response to therapy was reported in four trials, which included 64 patients in total. Of these, 11 patients developed an objective response (17.19%), and 1 patient achieved a mixed response (1.56%).

Four clinical trials including 63 patients reported numeric data on adverse effects, totaling 966 cases (15.33 AEs/patient). Morgan et al. [42] reported that no toxicities were attributed to the gene-marked cells. Duinkerken et al. [59] presented a case report on a patient who experienced subacute bilateral sensorineural hearing loss after receiving anti-MART-1-targeted TCR-T therapy. The most common adverse effects were general impairments including muscle pain (3.25 AE/patient), blood and lymphatic system disorders (3.56 AE/patient), and metabolism and nutrition system disorders (1.62 AE/patient).

Fifty-two immune-related adverse events were reported (0.64 AE/patient), including uveitis (*n* = 13, 16.05%), hearing impairment (*n* = 15, 18.52%), vitiligo (*n* = 19, 23.46%), and cytokine release syndrome (*n* = 5, 6.17%). These events are distributed across the subgroups as follows: 35 events in the DMF5 trials, 14 in the 1D3HMCys trial, 2 in the C-Cure 709 trial, and 1 in the case report in which the receptor was not specified.

The trials evaluating TCR-T therapy varied by the type of T-cell receptor employed.

#### 3.6.1. DMF5

Two trials (Johnson et al. [NCT00509288] [38] and NCT00706992) employed T cells transduced with the MART-1-specific F5 (DMF5) TCR in a total of 37 patients. Trial NCT00706992 did not report an objective response to therapy; in the other trial, comprising 20 patients, 6 patients developed an objective response, yielding an objective response rate of 30%. Adverse events were estimated at 604 cases across the 37 patients of the two trials (16.3 AE/patient). Thirty-five immune-related AEs were reported, all of them in the trial by Johnson et al., corresponding to 1.75 events per patient in that trial (35 events among 20 patients).

#### 3.6.2. 1D3HMCys

Rohaan et al. [24] (NCT02654821) utilized TCR-T therapy with T cells transduced with a modified MART-1-specific 1D3 TCR (1D3HMCys) in 11 patients. Two objective responses were observed (18%). Adverse events were estimated at 276 cases (25.1 AEs/patient), including uveitis (*n* = 2), vitiligo (*n* = 3), hearing impairment (*n* = 4), and cytokine release syndrome (*n* = 5); thus, the frequency of immune-related AEs was 1.27 per patient. In one patient who developed cytokine release syndrome, a fatal outcome characterized by neurologic damage and multiple organ failure was described by van den Berg et al. [40].

#### 3.6.3. C Cure 709 T-Cell Line

One trial by Køllgaard et al. [16] tested an allogeneic irradiated continuous T-cell line (C-Cure 709) expressing anti-MART-1 TCR in 15 patients. One patient developed an objective response (6.7%). Adverse events were estimated at 86 cases (5.73 AE/patient). Two patients developed vitiligo; thus, the frequency of immune-related AEs was 0.13 per patient.

The T-cell receptor was not specified in the trial by Morgan et al. [42] or in the case report by Duinkerken et al. [59]; these two records account for the remaining 18 patients of this group (*n* = 17 and *n* = 1, respectively). The report by Seaman et al. [39] describes audiovestibular toxicity observed in patients of NCT00509288 and is therefore not a separate trial.

The 1D3HMCys receptor was associated with the highest frequency of adverse events, including one fatal outcome following cytokine release syndrome. The highest objective response rate (30%) was observed for the DMF5 receptor.

### 3.7. Peptide Vaccines

Peptide vaccines represented the largest group of immunotherapies, comprising 19 studies with a total of 700 treated patients.

Objective responses to therapy were reported in nine trials, which included 111 patients evaluable for response by RECIST. Of these, seven patients developed an objective response (6.3%). Twenty-six patients also achieved stable disease (23.42%). The trial by Speiser et al. [27] contributed one of these objective responses (1 of 15 evaluable patients, 6.7%), and the trial by Jäger et al. [29] contributed three (three of three evaluable patients); the latter value rests on a single three-patient cohort and should not be read as a comparative result.

Fourteen clinical trials, comprising 649 patients, reported numeric data on AEs, totaling 5593 cases (8.62 AE/patient). The most common adverse events were injection site reactions (2.94 AE/patient), general and muscle pain (2.64 AE/patient), gastrointestinal disorders (0.67 AE/patient), neurologic AEs (0.58 AE/patient), and dermatologic AEs (0.55 AE/patient). Five cases of skin depigmentation (0.73%) and three cases of hearing impairment (0.44%) were observed among the 682 patients of the trials that reported adverse event data; the frequency of immune-related AEs was 0.01 per patient.

The clinical trial by Reynolds et al. [60] employed peptide vaccination with a pool of melanoma-associated antigens derived from shed material of melanoma cell lines. This study found induction of a T-cell response against MART-1 and MAGE-A3 antigens.

#### 3.7.1. MART-1 Epitope Variants

The peptide vaccines differed by the type of MART-1 epitope.

- **MART-1 27–35 (27L)**. One trial (NCT00059475) assessed vaccines based on the MART-1 epitope with leucine substitution (MART-1 27–35 (27L)) in two cohorts of 58 patients. The objective response was not reported. Adverse events were estimated at 195 cases (3.36 AE/patient).
- **MART-1 26–35 (27L) + MART-1 26–35**. Two trials (one arm of NCT00112242, Baumgaertner et al. [61], *n* = 19, and NCT01308294, Legat et al. [62], *n* = 16) assessed vaccines based on the decameric MART-1 peptide unmodified (MART-1 26–35) and with leucine substitution (MART-1 26–35 (27L)) in a total of 35 patients. None of the four patients evaluable for a response in these two trials achieved an objective response. Adverse events were estimated at 1536 cases (43.89 AE/patient).
- **MART-1 26–35 (27L)**. Eight trials (Ayyoub et al. [63], Di Pucchio et al. [64], Tarhini et al. [28] [NCT00471471], Lienard et al. [65], Urbani et al. [66], Baumgaertner et al. [NCT00112242] [61] (two arms, 20 patients in total), NCT00059475, and Nisticò et al. [67]) assessed vaccines based on the MART-1 26–35 (27L) epitope in a total of 142 patients. Three trials comprising 35 patients evaluable for response by RECIST reported an objective response to therapy; among these, two patients developed an objective response (5.7%). Di Pucchio et al. stated that no grade 3/4 adverse events were observed; all but two patients experienced fatigue, fever, or anorexia, while Ayyoub et al. reported only minor local pain after injection. Other trials provided numeric data on AEs, totaling 1376 cases among 132 patients (10.42 AE/patient). Two cases of vitiligo and three cases of hearing impairment were reported among the 142 patients of this subgroup that provided countable adverse event data (1.4% and 2.11%, respectively); the frequency of immune-related AEs was 0.035 per patient.
- **MART-1 27–35**. Three trials (Wang et al. [68], Cormier et al. [69], and NCT00059475) assessed vaccines based on the MART-1 27–35 epitope in a total of 81 patients. The objective response was reported only in the trial by Cormier et al., but no patient achieved an objective response. Cormier et al. did not specify the exact number of adverse effects but stated that toxicities were limited to local irritation at the injection site. Among other two trials, the estimated number of adverse events totaled 192 cases among 58 patients (3.31 AE/patient).
- **MART-1 long peptide**. One trial by Goldinger et al. [NCT00651703] [70] assessed peptide vaccines based on the MART-1 long epitope 16–35 in 21 patients. No objective response was achieved. The estimated number of adverse events was 187 cases among 21 patients (8.9 AE/patient).

Other trials did not specify whether the MART-1 epitope was used.

According to the reported data, only a peptide vaccine incorporating the MART-1 26–35 (27L) epitope led to an objective response in the peptide vaccine trials, and immune-related adverse events were reported only in that subgroup. These epitope-level comparisons are exploratory: no trial compared epitope variants head to head, and the subgroups differ in adjuvant, patient population, cohort size and reporting practice.

Some peptide vaccines also incorporated other melanoma-associated epitopes.

#### 3.7.2. Additional Melanoma-Associated Antigens

- gp100

Three trials (NCT00059475, Di Pucchio et al. [64], and Nisticò et al. [67]) administered vaccines containing the MART-1 and gp100 antigens to a total of 51 patients. No patients achieved an objective response. Nisticò et al. also reported a median relapse-free survival of 212.8 days and a median overall survival of 881.6 days. Di Pucchio et al. did not provide numerical data on adverse effects; among the other two trials, adverse events were estimated at 135 cases among 44 patients (3.07 AE/patient).

- gp100, MAGE-3, NA17

One trial (NCT00515528) investigated peptide vaccination based on gp100, MAGE-3, and NA17 antigens in 17 patients. One patient achieved an objective response (5.9%). Adverse events were estimated at 87 cases (5.12 AE/patient).

- Gp100, tyrosinase

Four trials (NCT00084656, NCT01989572, trial by Tarhini et al. [28] [NCT00471471], and trial by Jäger et al. [29]) investigated peptide vaccination based on MART-1, gp100, and tyrosinase peptides administered to a total of 320 patients. NCT00084656 and NCT01989572 did not report a response to therapy; among the 24 patients evaluable for response in the other two trials, five developed an objective response (20.8%), three of them reported by Jäger et al. in a cohort of three patients. Tarhini et al. [28] (NCT00471471) also reported a median PFS of 56.9 days and OS of 407.9 days. Adverse events were reported in three trials, totaling 1998 cases among 317 patients (6.3 AE/patient).

- MAGE-A3, NY-ESO-1, NA-17A

One trial by Legat et al. (NCT01308294) [62] investigated peptide vaccination based on MART-1, MAGE-A3, NY-ESO-1, and NA-17A antigens in 16 patients. No patients developed an objective response. Adverse events were estimated at 609 cases (38.06 AE/patient).

- NA17.A2, MAGE-3

One trial (NCT01307618) investigated peptide vaccination based on MART-1, NA17.A2, and MAGE-3 antigens in 10 patients. The trial did not report an objective response to therapy. Adverse events were estimated at 35 cases (3.5 AEs/patient).

- NY-ESO

One trial by Urbani et al. [66] investigated peptide vaccination based on MART-1 and NY-ESO antigens in 34 patients. The trial did not report a response to therapy. Adverse events were estimated at 82 cases (2.41 AE/patient).

- NY-ESO-1b, MAGE-A10

One trial by Baumgaertner et al. (NCT00112242) [61] investigated peptide vaccination based on MART-1, NY-ESO-1b(A), and MAGE-A10 in two cohorts of 29 patients in total. The trial did not report an objective response. Adverse events were estimated at 1526 cases (52.62 AE/patient); this value, an order of magnitude above that of most other trials, reflects exhaustive recording of all adverse events irrespective of grade rather than a genuine difference in tolerability, and illustrates the limited comparability of the AE/patient metric across trials.

- Mixed

Reynolds et al. [60] immunized 15 patients with a mix of melanoma antigens derived from shed material released by four melanoma cell lines. The presence of MAGE-A3 and MART-1 antigens in the vaccine was confirmed by the Western blot method. The response specifically directed against MAGE-A3 or MART-1 was induced in 60% of patients and found to correlate with the clinical outcome. In responders to these antigens, median relapse-free survival equated to 15 months compared with non-responders (median 3 months). Adverse effects were not reported.

### 3.8. Combination Approaches

#### 3.8.1. ACT + ACT

Two studies tested combinations of ACT therapies, comprising 21 patients in total:

- **TCR-T + DC vaccine**. The trial NCT00910650 employed T cells transduced with a DMF5 T-cell receptor coupled with dendritic cell infusion in 13 patients. This study reported 452 adverse events (34.77 AEs/patient) with four cases of uveitis (30.77%) and one skin depigmentation (7.7%); the frequency of immune-related AEs was 0.38 AEs/patient. The AE categories with the highest frequencies were metabolism and nutrition disorders (4.77 AEs/patient), respiratory diseases (4.62 AEs/patient), and neurologic/psychiatric disorders (4.54 AEs/patient). This study did not achieve any clinical response; in all patients, either tumor recurrence or disease progression occurred within 3 years from the first drug administration. Compared with TCR-T therapy alone, the combination of TCR-T therapy with DC vaccines showed a higher frequency of adverse events.
- **Autologous cells + DC vaccine**. In a study published by Saberian et al. [37] (NCT00338377), eight patients were treated with a combination of autologous cells and dendritic cells. The study reported a total of 142 AEs for the cohorts receiving TIL+DC and TIL only (17.75 AEs/patient), among which one case of vitiligo (12.5%) was observed. The AE categories with the highest frequencies were blood and lymphatic system disorders (5 AEs/patient), cardiovascular disorders (3.63 AEs/patient), and gastrointestinal disorders (2.63 AEs/patient). Four patients developed an objective response to treatment (50%), and two patients had disease stabilization (25%). The study reported a median PFS of 178.85 days and median OS of 730 days for the TIL+DC cohort.

#### 3.8.2. ACT + Non-ACT

Five studies evaluated combinatory therapies comprising both non-ACT and ACT approaches in a total of 65 patients.

- Autologous cells + peptide vaccines

The clinical trial by Romano et al. (NCT00324623) [71] and NCT00001832 described the infusion of autologous cells with peptide vaccination in 30 patients in total. Five objective responses were reported in the NCT00001832 cohort (5 of 18 evaluable patients, 27.8%); across the two trials of this subgroup, the objective response rate was 5 of 30 (16.7%). The total number of AEs was 341 (11.37 AE/patient). The most common categories of AEs were blood and lymphatic system disorders (5.93 AEs/patient), metabolism and nutrition disorders (2.0 AEs/patient), and general disorders including muscle pain (1.5 AE/patient). Two immune-related AEs were observed: one case of hearing impairment (3.33%) and one case of vitiligo (3.33%).

- TCR-T + peptide vaccines

Two studies combined TCR-T therapy with peptide vaccines, comprising 20 patients. Trial NCT00706992 did not report an objective response to therapy, while in the other trial (NCT00923195), no patient achieved an objective response. Adverse events occurred in 99 cases (4.95 AEs/patient). The most common groups of adverse events were blood and lymphatic system disorders (1.2 AEs/patient), nutrition disorders (0.65 AEs/patient), and infections and infestations (0.65 AEs/patient). Five cases of immune-related adverse events were reported in total (0.25 AEs/patient), including two cases of vitiligo (10%), one case of uveitis (5%), and two cases of hearing impairment (10%). The two studies are the peptide vaccine arms of NCT00706992 (Arms II and IV: F5 T cells with the MART-1 26–35 (27L) peptide vaccine, with or without IL-2; *n* = 16) and NCT00923195 (*n* = 4).

- TCR-T + DNA vaccines

Two trials combined TCR-T therapy with DNA vaccines, comprising 15 patients. Only trial NCT00612222 reported a clinical response, but no objective response was achieved. The total number of adverse events was 75 cases (5 AE/patient). The most common AE groups were blood and lymphatic system disorders (1.93 AE/patient) and dermatologic toxicity (0.67 AE/patient). Three cases of autoimmunity (0.2 AE/patient) were reported, including one case of uveitis (6.7%), one case of vitiligo (6.7%), and one case of hearing impairment (6.7%). These are two distinct registrations that used the same investigational cell product: NCT00706992 is a multi-arm trial in which Arms V and VI combined F5 T cells with an ALVAC MART-1 vaccine, with or without IL-2 (*n* = 11), whereas NCT00612222 is a separate trial in which F5 cells were administered with an ALVAC vaccine and IL-2 after cyclophosphamide and fludarabine (*n* = 4).

Among the TCR-T-based approaches, the combinations with peptide vaccines and with DNA vaccines showed the lowest adverse event rates (4.95 and 5.0 AE/patient) and the lowest frequency of immune-related adverse events (0.25 and 0.2 events per patient). Each of these figures rests on two small trials and is not a comparative result.

#### 3.8.3. Non-ACT + Non-ACT

- Peptide vaccines + DNA vaccines

One trial by Ribas et al. (NCT00688090) [72] investigated the MKC1106-MT therapeutic vaccine in 18 patients. The vaccine comprised recombinant plasmid encoding fragments of MART-1 tyrosinase antigens, combined with peptide analogues of MART-1 (26–35) and tyrosinase (369–377) epitopes with norvaline substitutions. No patient developed an objective response to therapy. Adverse events were estimated at 19 cases (1.06 AE/patient). The most frequent group of AEs was general impairments (0.61 AEs/patient). No immune-related adverse effects were observed.

## 4. Discussion

Clinical trial registries and bibliographic databases (PubMed, Scopus) were systematically searched for clinical trials of immunotherapies targeting MART-1-expressing tumors. Extracted data included objective response rates, median survival, and adverse event incidence and profiles. Finally, a comparative evaluation of the safety and efficacy profiles across different immunotherapy types based on the accessible clinical trials was performed. The main findings of the systematic review are presented below.

In the host, cytotoxic T cells continuously survey cell membranes for self-peptides and peptides derived from infection or malignant transformation, presented in the context of MHC-I molecules [73]. The intrinsic molecular machinery responsible for peptide translocation, proofreading by chaperones, and loading onto the MHC-I molecules is called the peptide loading complex [74]. However, tumor cells exposing altered epitopes alone are often insufficient to activate cytotoxic T cells; to initiate a potent antitumor cytotoxic response, naive progenitors must encounter professional antigen-presenting cells (APCs), which prime lymphocytes with an epitope of 9 to 11 amino acids in length within the MHC-I molecule during a process referred to as cross-presentation [75]. These epitopes are generated after APCs internalize tumor antigens or whole virally infected and transformed cells, following antigen proteolysis and subsequent binding to the MHC molecule [76]. The described antigen presentation machinery underpins distinct immunotherapy approaches aimed at tumor elimination [23].

The main principle of immunotherapy resides in imitation of distinct stages of the natural immune system response to the tumor development, from priming tumor-directed cells during cross-presentation carried out by APCs, to tumor recognition and subsequent killing mediated by cytotoxic cells. Cancer vaccines introduce a specific tumor antigen for endogenous presentation. The adoptive cell therapy strategies, on the other hand, involve administration of ex vivo-manipulated cells. These include epitope-loaded or genetically modified [77,78], as well as ready-to-act cells, either ex vivo-expanded autologous tumor-reactive lymphocytes or TCR-engineered T cells.

MART-1 is a tumor-associated antigen expressed abundantly in melanoma cells. This feature enables the induction of antitumor response-harnessing cytotoxic lymphocytes, either generated in vivo following cancer vaccination or produced ex vivo and infused to patients. Immunotherapy employs several MART-1-derived peptides comprising immunogenic epitopes, including those modified with amino acid substitution for improved recognition by CTLs (Table 3).

Immunotherapies targeting MART-1 may also affect healthy cells in the ear, eye, and skin, leading to off-tumor, on-target side effects such as sensorineural hearing loss, decreased vision, and vitiligo, a subset of adverse effects referred to as immune-associated in the present paper [39,59,79]. Different immunotherapy approaches are associated with various risks of such adverse effects and their severity in addition to immune challenges contributed by tumor adaptation that may compromise their efficacy.

Non-ACT immunotherapy approaches for treating MART-1-expressing melanoma include peptide and DNA vaccines, both of which lead to antigen processing and cross-presentation by DCs. Peptide-based vaccines utilize whole antigens or specific epitopes that must be taken up by APCs in vivo to prime naive T cells, leading to their expansion and subsequent antitumor effects. In the case of DNA vaccines, antigen processing is shaped by the route of administration. Intramuscular administration of a DNA vaccine results in transfection of myocytes; antigen-presenting cells (APCs) either migrate to the injection site and cross-present the antigen or become directly transfected [80]. An alternative injection route is intranodal, which provides a site of immune cell accumulation where the induction of an antitumor cytotoxic T-cell response is initiated [81].

For DNA vaccines, antigen introduction can be achieved using either viral vectors or plasmid vectors. The described studies used either the replication-deficient vector ALVAC or recombinant vaccinia virus that had been treated with psoralen and long-wave UV. Replication-deficient viral vectors are safer alternatives to replicating vectors, although they may not be as potent in inducing immunity [82]. Plasmid-based vaccines represent a more flexible platform, although their transfection efficacy and immunogenicity are lower [80]. In the current systematic review, DNA vaccines in general demonstrated good safety; however, the higher incidence of adverse effects was observed for plasmid vector-based vaccines compared to viral vector vaccines. One case of grade 1 vitiligo was observed by Adamina et al. during treatment with recombinant vaccinia virus comprising several melanoma-associated epitopes fused to CD80 and CD86 costimulatory molecules; moreover, in this trial, the patient who developed skin depigmentation had relatively longer survival despite the advanced disease [31]. This trial, together with the trial by Rosenberg et al. employing an adenoviral vaccine, achieved six objective responses in total, whereas, for plasmid-based vaccines, the number of objective responses was either not reported or no responses were achieved. Thus, in the present review, objective responses in the DNA vaccine group were recorded only in the viral vector trials; with three viral vector and three plasmid trials, this is a descriptive observation rather than evidence of superior efficacy.

Peptide-based vaccine therapy is often reported to induce a delayed immune response and inhibit tumor growth, but it does not cause significant tumor shrinkage [83]. Peptide vaccines are often described as having favorable safety profiles; nevertheless, when calculating the frequency of adverse events per patient, peptide vaccines demonstrated safety comparable to that of ACT approaches. However, across the 19 peptide vaccine trials, the number of grade III-IV AEs was estimated at only 21 cases. These severe AEs were restricted to thrombosis, granulocytopenia, fatigue, and lab abnormalities. The unexpectedly high frequency of AEs may be due to researchers recording all AEs regardless of their severity, including those typically considered negligible.

Among both groups of clinical trials evaluating DNA and peptide vaccines, some vaccines incorporated other melanoma-associated epitopes, such as gp100, tyrosinase, and MAGE-A3. Unfortunately, the contribution of these additional epitopes cannot be evaluated due to the absence of clinical trial designs that explicitly compare the efficacy of single-epitope versus multi-epitope vaccines. In a cross-trial comparison, higher objective response rates were reported for multi-epitope vaccines, although the trials differ in every other respect and the observation cannot be interpreted as an effect of the additional epitopes. Targeting several antigens may be beneficial, as it potentially allows for overcoming tumor heterogeneity. Multi-targeted immunotherapy strategies have also been considered by other research groups [84]. Cross-trial comparison also revealed that vaccines containing the MART-1 decamer with leucine substitution were the only peptide vaccines for which an objective response was recorded, whereas in the DNA vaccine trials, the single objective response was obtained with the unmodified nonamer. With one to three trials per epitope, these differences cannot be interpreted as an effect of the epitope itself.

Adoptive cell therapies, on the other hand, use the infused cells either to produce an antigen-directed response in the host by presenting a certain antigen or to eliminate cells exposing the antigen. The described strategies comprised the infusion of DCs, lymphocytes either isolated and expanded from the populations of TIL or PBL or genetically modified to express T-cell receptors with desired specificity.

DC vaccines manipulate the interplay between innate and adaptive immunity. Dendritic cells can induce a response by presenting specific antigens to naive T cells in draining lymph nodes, leading to the generation of active helper and cytotoxic T cells [85]. Furthermore, DCs suppress regulatory T-cell (Treg) activation and promote cytotoxic responses via pro-inflammatory cytokines (TNF-α, IFN-α/β, and IL-12p40/IL-12p70). These cytokines exhibit anti-neoplastic characteristics in a feedback manner and support the expansion of melanoma-specific CTLs [86].

The mode of loading in DC-based cancer vaccines determines how the antigen is going to be processed and presented by APCs, which may affect vaccine efficacy. Peptide loading has the advantage of not being restricted to a certain HLA haplotype, as one peptide may give different epitopes with a distinct affinity to different HLA haplotypes. Loading dendritic cells with oncolysate may hinder antigen processing and presentation due to the presence of unrelated epitopes. In contrast, loading with a certain epitope retains predefined specificity. Trakatelli et al. showed that dendritic cells transfected with ALVAC virus encoding MAGE-1/MAGE-3 activated cytotoxic cells more efficiently than peptide-loaded dendritic cells [87]. However, based on the available data, a higher frequency of immune-related AEs was reported in the two trials of virally transduced DC vaccines than in the other DC subgroups. Tsao et al. [25] published a case report on three patients with advanced metastatic melanoma who developed leukoderma during treatment; despite evidence of melanocyte destruction, no response was achieved.

However, the efficacy of cancer vaccines, including those based on DNA, peptides, and dendritic cells, is often compromised by the immunosuppressive tumor microenvironment. The tumor microenvironment may comprise cells that secrete immunosuppressive cytokines or express immune checkpoints impairing dendritic cell function [88,89]. Strategies based on cells with direct cytotoxic activity bypass the need for priming T cells against certain antigens, which potentially could limit the influence of the tumor immunosuppressive mechanisms that target antigen processing or antigen presentation. Such approaches for treating MART-1-expressing melanomas include TCR-T therapy and infusion of autologous cells, derived either from the pool of tumor-infiltrating lymphocytes or from peripheral blood mononuclear cells. In either case, described therapies are associated with distinct strengths and limitations.

Autologous cells-based therapies employ a polyclonal, naturally forming, tumor-targeted CTL repertoire, covering a wide range of antigens, which reduces the risk of tumor immune evasion through antigen downregulation. Tumor-specific cells can either be extracted from the pool of tumor-infiltrating lymphocytes or peripheral blood mononuclear cells. The study by Oliveira et al. demonstrated that melanoma-specific cytotoxic T cells primarily accumulate in the tumor microenvironment [90]. In 27% of patients, only tumor-infiltrating lymphocytes (TILs) were responsive to tumor stimulation, in contrast to cytotoxic cells extracted from the peripheral blood, despite their antigen-experienced phenotype. This finding has been interpreted as indicating that TIL populations harbor a greater proportion of tumor-reactive cytotoxic cells [91].

However, this type of therapy faces technical challenges, such as prolonged ex vivo culture and an invasive procedure for cell harvesting, which is distressing and may not be feasible for some patients [92]. Heterogeneity of the retrieved T-cell product may compromise a clinical benefit, as the response to TIL therapy showed a strong correlation with immunotype features of infused T cells [93]. Some T cells in the tumor environment may possess an exhausted phenotype or be terminally differentiated, which limits their cytolytic antitumor activity and in vivo persistence [7,94]. A study by Van Oijen et al. showed that the high frequency of MART-1-specific cells exhibiting a memory or effector phenotype in the peripheral blood of advanced melanoma patients does not confer a survival benefit. The absence of vitiligo in these patients further suggests a lack of functionality in these T cells [95]. Another possible explanation for the absence of melanocyte destruction following TIL infusion lies in the inferior avidity of naturally generated T cells responsive to tumor-associated antigens [96]. These challenges necessitate modifications to TIL therapy, including screening for various exhaustion markers and strategies to enhance survival and tumor-homing ability [97].

TCR-T therapy, on the other hand, ensures a consistent monoclonal product with a desirable phenotype, antigen specificity, and T-cell receptor affinity; the restriction on TCR specificity requires careful consideration of patient eligibility, conducting treatment only in patients with confirmed antigen expression and an appropriate haplotype.

Targeting of healthy cells occurs due to the expression of identical antigens, a phenomenon known as an off-tumor, on-target effect [98]. The presence of MART-1-expressing cells in the ear, eye, and skin causes immune-related adverse effects, including sensorineural hearing loss, decreased vision, and skin depigmentation. TCR-T therapy employs T cells expressing TCRs with improved affinity, which is associated with an enhanced risk of off-target effects. This has been proposed as an explanation for the higher frequency of immune-related adverse events reported in the TCR-T trials than in the autologous cell transfer trials, which use cytotoxic cells with an affinity in the natural range. Use of bispecific TCR-like antibodies offers a means to overcome immune cell hyperactivation. By simultaneously binding the pMHC complex on the tumor surface and CD3 on the T-cell surface, they promote the formation of the immunologic synapse. Careful tuning of the tumor antigen and CD3 affinity of TCR allows controlled immune system activation and precise tumor-directed toxicity [3].

Researchers proposed various T-cell receptor structure solutions, with varying degrees of benefit, to optimize the risk–efficacy ratio of anti-melanoma TCR-T therapy. For example, treatment with a 1D3HMCys T-cell receptor comprising murine constant parts showed an unfavorable safety profile, causing several cases of autoimmune-related AEs, including severe cases of cytokine release syndrome, resulting in death. In contrast, the well-described T-cell receptor DMF4 and the affinity-enhanced DMF5 targeting MART-1 26/27–35 epitopes successfully induced clinical responses in patients. DMF5 was reported to confer a greater clinical benefit but also an elevated risk of autoimmunity [99], which aligns with our findings (1.75 immune-related events per patient in the trial by Johnson et al.). Structures and binding modes of these receptors were extensively characterized [100] and continue to be model T-cell receptors for various research applications [101,102]. Moreover, attempts to produce an “off-the-shelf” product for TCR-T therapy resulted in the generation of irradiated C-Cure 709 T-cell line transduced with a MART-1-specific T-cell receptor.

Table 4 summarizes MART-1-targeting TCRs described in the literature.

Extensive studies on the structural characteristics of T-cell–pMHC interactions along with efforts to generate T-cell receptors with improved affinity and target precision reflect the general interest in this approach.

Some research groups attempted to implement combination strategies of autologous T-cell infusion and DC vaccines with other cell-based therapies. Numerous studies in murine models demonstrated that such regimens are superior in augmenting the effector-to-regulatory T-cell ratio and improving the potency and robustness of the antitumor immune response [44,106,107].

Lou et al. demonstrated that autologous cell infusion and dendritic cell vaccination in murine models act in a complementary way, enhancing the robustness of the antitumor response relative to each treatment [108]. Saberian et al. therefore attempted co-administration of TILs containing 0.1% MART-1-reactive T cells with DC pulsed with MART-1 (26–35 (27L) peptide, but the vaccination did not result in enhanced T-cell persistence [37]. The objective response rate achieved in the TIL+DC arm was better than that of the TIL arm (50% and 30%, respectively); these results lack statistical power due to the small sample size.

The trial by Chodon et al. tested a short ex vivo TCR transgenic cell manufacture protocol excluding a cryopreservation step together with MART 1 26–35 peptide-loaded DC vaccination [109]. Two patients developed acute respiratory distress with elevated cytokine levels one week after receiving TCR transgenic cells. These patients also had transient tumor responses with longer persistence of transferred T cells with evidence of in vivo expansion following the DC vaccine. Furthermore, one patient receiving the scheduled treatment developed a recall whole-body rash and re-expansion of TCR transgenic T cells after DC vaccination, supporting the idea that DC vaccines contribute to in vivo activation of MART-1-specific T cells.

The combination strategy of TCR-T therapy with DC vaccination implemented by Chodon et al. [109] demonstrated a significantly worse safety profile than TCR-T therapy alone. According to the information provided from ClinicalTrials.gov (NCT00910650), the treatment resulted in a high frequency of immune-related AEs: four patients developed uveitis and one patient developed vitiligo, and two patients with severe respiratory distress had elevated cytokine levels; however, their levels were not as high as reported in life-threatening cases of CRS. The possible cause is a modified protocol excluding a cryopreservation step, which enabled improved T-cell persistence and expansion but also caused more toxicities. Saberian et al. reported adverse events for cohorts receiving autologous cells alone and in combination with dendritic cells together, without data separation [37]; therefore, the safety of the combination approach could not be assessed.

Combining ACT therapies with peptide and DNA vaccines may provide several advantages, such as reducing the immunosuppressive effects of the tumor microenvironment. Cancer vaccines can complement ACT by promoting immunostimulatory effects. Antigens used for in vitro CTL stimulation can be developed into vaccines to enhance in vivo cytotoxic responses [110,111]. Autologous cell infusion with peptide vaccination was evaluated in two clinical trials, in which the overall adverse event rate was higher than in autologous cell transfer alone (11.37 versus 9.25 AE/patient), and two immune-related events were recorded among 30 patients. With two trials and no common comparator, these figures do not support an inference about the comparative safety of the combination.

The combination of TCR-T therapy with DNA and peptide vaccines was presented in three trials. Based on available evidence, among TCR-T therapy-based approaches, the combination of TCR-T therapy with DNA vaccination was associated with the lowest frequency of immune-related adverse events (0.2 versus 0.25 events per patient for the combination with peptide vaccines), although the overall adverse event rates of the two combinations were similar and each rests on two small trials.

The present systematic review has certain limitations. The included studies differed substantially in trial phase, cohort sizes, patient characteristics, treatment protocols, and endpoints assessed. The broad inclusion of heterogeneous studies was intentional, allowing the review to capture relevant clinical evidence that would otherwise have been excluded by restricting eligibility to closely matching study designs and reporting formats. This approach enabled a broader descriptive comparison of therapeutic strategies, although the resulting patterns cannot be interpreted as evidence of comparative efficacy or safety. The inconsistencies identified also highlight the need for more consistent outcome definitions and reporting practices to support future evidence synthesis. No formal risk-of-bias assessment of the included studies was performed: the trials are predominantly single-arm and early-phase, several records were available only as registry entries, and the information required by standard instruments is not reported for most of them. It also should be noted that the search was limited to Scopus, PubMed, ClinicalTrials.gov, and EU CTR, and did not include Embase, Web of Science, or WHO ICTRP, which may have resulted in the omission of some relevant studies. Concomitant treatments (IL-2, immunosuppressive regimens, and adjuvants) could confound clinical benefit and safety, making it impossible to assess the exclusive contribution of MART-1-targeted immunotherapy. Certain immunotherapy modalities were underrepresented and therefore provided limited data, precluding meta-analysis. Variability in melanoma patient characteristics across trials, including differences in melanoma localization, stage, presence of metastasis and overall severity of disease, further complicated comparison. A small number of included trials involved patients with ocular melanoma, which is biologically distinct from cutaneous melanoma. No separate analysis was performed due to the limited number of such cases. In addition, there was a significant heterogeneity in adverse event reporting: some trials reported the number of AEs, whereas others reported the number of patients affected by AEs; some trials provided overall AE counts without cohort separation; and some groups reported adverse events they considered to be treatment-related, while others presented data on all AEs without stratification. Given this heterogeneity, the AE/patient frequencies should be interpreted with caution, and the descriptive comparisons are provided for overview purposes only. One report identified as potentially eligible, the pilot trial of a MART-1a peptide vaccine by Block et al. [26], could not be retrieved in full text and was excluded for this reason; its outcomes are therefore not represented in the pooled figures for peptide vaccines. No meta-analysis and no formal comparative efficacy analysis were performed, and all comparisons presented here are descriptive. No clinical trial of an ImmTAC directed at a MART-1 epitope was identified, so this platform is not represented among the included studies. Standardized reporting of outcomes should be considered to strengthen future evidence and improve analytical robustness.

## 5. Conclusions

Among immunotherapies targeting MART-1-expressing melanomas, higher objective response rates and a higher frequency of immune-related adverse events, including severe cases of cytokine release syndrome, were reported in the autologous cell transfer and TCR-T trials than in the vaccine trials. This is an uncontrolled cross-trial observation rather than a comparative result, and it is driven by the autologous cell transfer and TCR-T trials: the objective response rate reported in the dendritic cell vaccine trials was of the same order as in the peptide and DNA vaccine trials. In this group of therapies, TCR-T therapy represents a promising approach characterized by continuous research on development of MART-1-restricted T-cell receptors capable of inducing a potent and precise anti-melanoma immune response, although careful safety considerations are required. In contrast, peptide and DNA vaccines encouraging the body to elicit MART-1-targeted immune responses offer a favorable safety profile, characterized by a minimal risk of autoimmune reactions, and potentially may serve as a complementary therapy for cell-based regimens. It should be noted that adoptive cell therapies were typically administered with lymphodepletion and IL-2, which may have contributed to both efficacy and toxicity; therefore, the observed differences between modalities cannot be attributed solely to MART-1 targeting.

MART-1 is well-studied and a promising immunotherapeutic target, owing to its abundant expression in melanoma cells and potential immunogenicity, which is supported by favorable clinical trial outcomes. Ongoing research into MART-1-targeting immunotherapies may broaden therapeutic options for melanoma patients, although the clinical evidence remains heterogeneous and further studies are needed. Future investigations could focus on refining specificity to improve tumor cell eradication while lowering the risk of off-target effects, and on strategies for managing immunotherapy-associated adverse events.

## Figures and Tables

**Table 1:** Search strings used in each bibliographic database and clinical trial registry and the respective number of records retrieved. The last search was performed on 15 January 2026.

Database	Search Query	No. of Results
ClinicalTrials.gov	(“Melan-A” OR “MART-1” OR “MART1” OR “melanoma antigen recognized”) AND (“melanoma” OR “skin cancer”)	85
Scopus	TITLE ((trial OR study OR clinical OR treatment) AND (MART-1 OR MART1 OR (melanoma antigen recognized)))	317
EU Clinical Trials Register	MART-1 OR Melan-A	8
PubMed	(“MART-1”[Title] OR “MART1”[Title] OR “melanoma antigen recognized”[Title]) AND (trial[Title] OR study[Title] OR clinical[Title] OR treatment[Title])	25

**Table 2:** Table of clinical trials that achieved objective response to therapy. Each trial is represented by the first author of corresponding publication and/or NCT number. For each trial, the color and size of the circular diagram vary according to the objective response rate and sample size. Larger size of circular diagram indicates larger sample size. The color scale for objective response rate is provided. ORR—objective response rate; NR—not reported, NS—not stated; PFS—progression-free survival, OS—overall survival. “Patients evaluable for response” are the patients of a given cohort who entered the trial with measurable disease assessable by RECIST. Only these patients contribute to the denominator of the objective response rate. “Data source” indicates whether the outcome data were taken from a peer-reviewed publication, from a trial registry record for which no corresponding full-text publication could be identified, or from a single-patient case report. Survival values are medians in days, extracted as reported or, where only individual patient data were available, estimated by the Kaplan–Meier method.

Clinical Trials Overview—ORR (*n*/*N*) > 0 ■ 0% ■ 0.1–10% ■ 10–30% ■ >30%									
Trial	Therapy	Data Source	Start Year	Phase	Patients Treated (*n*)	Patients Evaluable for Response (*n*)	Survival (Median, Days)	ORR (*n*/*N*)	Diagram
**Non-adoptive cell immunotherapy**									
**Peptide vaccines**									
Speiser et al. [27] (PMID: 20842051) 3 sub-studies: NCT00306514 NCT00306566 NCT00306553	MelQbG10 (MART-1 16–35 (A27L) + CpG-ODN) + Montanide ISA-51	Publication + registry record	2006	I–II	22	15	NR	6.7% (1/15)	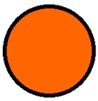
NCT00515528	Arm I: MART-1, gp100, MAGE-3, NA17, GM-CSF in Montanide; Arm II: MART-1, gp100, MAGE-3, NA17, GM-CSF in Montanide peptide vaccine + Ontak.	Registry record only	2007	II	17	17	NR	5.9% (1/17)	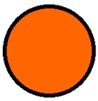
NCT00471471 Tarhini et al. [28] (PMID: 22495394)	MART-1 (26–35, 27L), gp100 (209–217, 210M), tyrosinase (368–376, 370D), combined with the GM-CSF and PF3512676, emulsified in Montanide ISA.	Publication + registry record	2008	I	21	21	PFS: 56.9 OS: 407.9	9.5% (2/21)	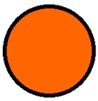
Jäger et al. [29,30] (PMID: 8690525, 11920589)	Peptide vaccine with MART-1-derived peptides, tyrosinase, and gp100.	Publication	1995	NS	3	3	NR	100% (3/3)	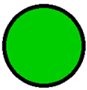
**DNA vaccines**									
Adamina et al. [31] (PMID: 19935776) NCT00116597	Recombinant vaccinia virus encoding gp100 280–288, MART-1 27–35, tyrosinase 1–9, and CD80/CD86 + GM-CSF.	Publication + registry record	2002	I–II	15	4	PFS: 310 OS: 488.5	25% (1/4)	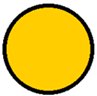
Rosenberg et al. [32] (PMID: 9862627)	Recombinant adenovirus encoding MART-1 with or w/o IL-2.	Publication	NS	I	36	36	NR	13.9% (5/36)	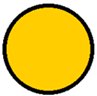
**Adoptive cell immunotherapy**									
**Autologous cell transfer**									
NCT00001832	Phase I: Cyclophosphamide, Fludarabine + Cells intravenous with or w/o IL-2; Phase II: Cyclophosphamide, Fludarabine + Cells intravenous or intraarterial with or w/o IL-2 + with or w/o G-CSF.	Registry record only	1999	II	89	89	NR	25.8% (23/89)	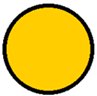
NCT00720031 Khammari et al. [33] (PMID: 19554023)	Dacarbazine + autologous MART-1 A27L-reactive PBMCs + INF-alpha + IL-2.	Publication + registry record	2000	I–II	14	14	NR	42.9% (6/14)	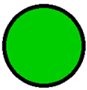
Vignard et al. [34] (PMID: 16177129)	Tumor-reactive MART-specific T-cell clones + IL-2 + INF-alpha.	Publication	NS	I–II	10	10	NR	10% (1/10)	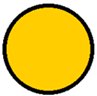
Dudley et al. [35] (PMID: 12242449)	Cyclophosphamide and fludarabine + tumor-reactive T cells + IL-2.	Publication	NS	NS	13	13	NR	46.2% (6/13)	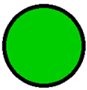
Meidenbauer et al. [36] (PMID: 12574389)	MART-1-specific CD8+ T cells + IL-2.	Publication	NS	NS	8	8	NR	12.5% (1/8)	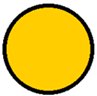
Saberian et al. [37] (PMID: 34021033) NCT00338377	MART-1 reactive CD8+ TIL.	Publication + registry record	2006	II	10	10	OS: 1496.5 PFS: 94.9	30% (3/10)	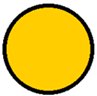
**TCR-T therapy**									
NCT00509288 Johnson et al. [38] (PMID: 19451549) Seaman et al. [39] (PMID: 22597578)	Cyclophosphamide + Fludarabine + Anti-MART-1 F5 TCR transduced TILs/PBLs + IL-2.	Publication + registry record	2007	II	20	20	NR	30% (6/20)	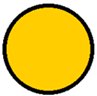
NCT02654821 Rohaan et al. [24] (PMID: 35865122) van den Berg et al. [40] (PMID: 25896248)	Cyclophosphamide + fludarabine + IL-2 + 1D3HMCys TCR transduced PBLs.	Publication + registry record	2008	I–II	11	11	PFS: 85 OS: 222	18.2% (2/11)	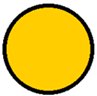
Køllgaard et al. [16] (PMID: 19530030) Duval et al. [41] (PMID: 16489078)	C-Cure 709 cells administered intratumorally.	Publication	2002	I	15	15	NR	6.7% (1/15)	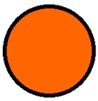
Morgan et al. [42] (PMID: 16946036)	Lymphodepletion + PBLs transduced with anti-MART-1 TCR.	Publication	NS	NS	17	17	NR	11.8% (2/17)	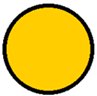
**Dendritic cell vaccines**									
Panelli et al. [43] (PMID: 10916759)	DCs pulsed with MART-1 (27–35), DCs pulsed with gp-100–209-2M with or w/o IL-2.	Publication	NS	I	7	7	NR	14.3% (1/7)	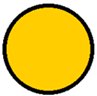
Butterfield et al. [44] (PMID: 12631598)	MART-1 27–35 peptide-pulsed autologous DCs intradermally or intravenously.	Publication	NS	I	18	10	NR	10% (1/10)	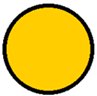
Ribas et al. [45] (PMID: 15314544)	MART-1 27–35 peptide-pulsed DCs intradermally.	Publication	NS	II	9	4	NR	25% (1/4)	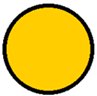
Palucka et al. [46] (PMID: 16971810)	Autologous monocyte-derived DCs loaded with killed allogeneic Colo829 melanoma cell line.	Publication	2002	I	20	20	OS: 684	10% (2/20)	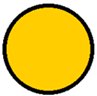
NCT00672542 Dannull et al. [47] (PMID: 23934126)	MART1, MAGE-3, gp100, and tyrosinase RNA–transfected DCs derived from iP siRNA–transfected monocytes.	Publication + registry record	2008	I	12	2	NR	100% (2/2)	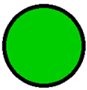
**Combination (ACT + ACT)**									
Saberian et al. [37] (PMID: 34021033) NCT00338377	MART-1 reactive CD8+ TIL + DCs pulsed with MART-1 peptide MART-1 26–35 (27L) + IL-2.	Publication + registry record	2006	II	8	8	PFS: 178.85 OS: 730	50% (4/8)	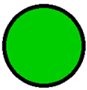
**Combination (ACT + non-ACT)**									
NCT00001832	Cyclophosphamide + fludarabine + IL-2 + GMSF + gp100:209–217(210M) (in gp100-reactive patients) + MART-1:26–35 (27L) (in MART-1-reactive patients).	Registry record only	1999	II	18	18	NR	27.8% (5/18)	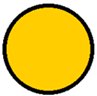

**Table 3:** MART-1 peptides employed in immunotherapy. The response rate given for each peptide is the highest value reported in an individual clinical trial and is calculated on the patients of that trial who were evaluable for response by RECIST. One to three trials are available per epitope, so the values are not comparable across rows.

Name	Sequence	Best Objective Response Rate Reported in an Individual Clinical Trial (%)	Clinical Trials (NCT and Refs.)
MART-1 26–35 (27L) (modified)	ELAGIGILTV	Peptide vaccines: 9.5% (Tarhini et al., NCT00471471; 2/21) DNA vaccines: 0% (Geukes Foppen et al.; 0/9)	Peptide vaccines: [28,63,64] (NCT00471471), [61,65,66] (NCT00112242), [67], NCT00059475 DNA vaccines: [48]
MART-1 27–35 (native)	AAGIGILTV	Peptide vaccines: 0% (Cormier et al.; 0/23) DNA vaccines: 25% (Adamina et al., NCT00116597; 1/4)	Peptide vaccines: NCT00059475, [68,69] DNA vaccines: [31]
MART-1 27–35 (27L) (modified)	LAGIGILTV	Peptide vaccines: not reported	Peptide vaccines: NCT00059475
MART-1 16–35 (long epitope)	GHGHSYTTAEEAAGIGILTV	Peptide vaccines: 0% (Goldinger et al.; 0/14)	Peptide vaccines: [70]
MART-1 16–35 (27L)	GHGHSYTTAEELAGIGILTV	Peptide vaccines: 6.7% (Speiser et al.; 1/15)	Peptide vaccines: [27]
MART-1 26–35 (native)	EAAGIGILTV	Peptide vaccines: 100% (Jäger et al.; 3/3)	Peptide vaccines: [29]

**Table 4:** Table of MART-1 targeting T-cell receptors with the specified binding affinity (KD) to the MART-1-derived epitopes complexed with the HLA-A2 molecule. Ref. = reference.

TCR	Affinity, KD	Description	Ref.
DMF4	KD = 29 ± 4 μM (HLA-A2 EAA) KD = 170 ± 11 μM (HLA-A2 AA)	- Cross-reacts with nonameric and decameric variants of MART-1 epitope via spatial reorientation toward MHC–peptide complex; - Clinical trial reported an objective response rate of 11.8%.	[99,100]
DMF5	KD = 5.6 ± 1.5 μM (HLA-A2 EAAGIGILTV) KD = 40 ± 2 μM (HLA-A2 AAGIGILTV)	- Features permissive architecture enabling equal binding mode to nonameric and decameric peptides; - Exhibit higher affinity than DMF4; - Achieved 30% cancer regression rate in clinical trials, but caused eye, ear and skin autoimmune toxicity.	[99,100]
MEL5	KD = 18 μM (HLA-A2 ELAGIGILTV)	- Superior recognition for naturally expressed nonapeptide.	[103]
α24β17	KD = 0.6 μm (HLA-A2 ELAGIGILTV)	- TCR derived from natural MEL5 with enhanced affinity generated by the phage display method.	[104]
1D3HMCys	Reported not to be higher than DMF 5	- Gene-optimized high-affinity 1D3 TCR, obtained from a metastatic melanoma patient; - Comprises murine constant domains within the α and β chains to promote preferential pairing; - Contains a second interchain disulfide bridge via an additional cysteine pair; - Caused severe on-target, off-tumor toxicity and cytokine release syndrome and fatal outcome.	[104,105]

## Data Availability

The raw data supporting the conclusions of this article will be made available by the authors on request.

## Supplementary Materials

The following supporting information can be downloaded at https://www.mdpi.com/article/10.3390/cancers18183016/s1, Table S1: Characteristics and outcomes of the clinical trials included in the review.

## Abbreviations

The following abbreviations are used in this manuscript:

MART-1	Melanoma antigen recognized by T cells 1
TCR	T-cell receptor
ACT	Adoptive cell therapy
ORR	Objective response rate
AE	Adverse event
RFS	Relapse-free survival
OS	Overall survival
PFS	Progression-free survival
TTP	Time to progression

## References

[1] Garbe C., Amaral T., Peris K., Hauschild A., Arenberger P., Basset-Seguin N., Bastholt L., Bataille V., Brochez L., Del Marmol V. (2025). European consensus-based interdisciplinary guideline for melanoma. Part 2: Treatment—Update 2024. Eur. J. Cancer.

[2] Haanen J., Los C., Phan G.Q., Betof Warner A. (2024). Adoptive Cell Therapy for Solid Tumors: Current Status in Melanoma and Next-Generation Therapies. Am. Soc. Clin. Oncol. Educ. Book.

[3] Lopatnikova J.A., Sennikov S.V. (2025). Bispecific immunotherapy based on antibodies, T-cell receptors, and aptamers: Mechanisms of action, adverse effects, and future perspectives. Front. Immunol..

[4] Weber J.S., Carlino M.S., Khattak A., Meniawy T., Ansstas G., Taylor M.H., Kim K.B., McKean M., Long G.V., Sullivan R.J. (2024). Individualised neoantigen therapy mRNA-4157 (V940) plus pembrolizumab versus pembrolizumab monotherapy in resected melanoma (KEYNOTE-942): A randomised, phase 2b study. Lancet.

[5] Seth R., Agarwala S.S., Messersmith H., Alluri K.C., Ascierto P.A., Atkins M.B., Bollin K., Chacon M., Davis N., Faries M.B. (2023). Systemic Therapy for Melanoma: ASCO Guideline Update. J. Clin. Oncol..

[6] Teixido C., Castillo P., Martinez-Vila C., Arance A., Alos L. (2021). Molecular Markers and Targets in Melanoma. Cells.

[7] Yarza R., Bover M., Herrera-Juarez M., Rey-Cardenas M., Paz-Ares L., Lopez-Martin J.A., Haanen J. (2023). Efficacy of T-Cell Receptor-Based Adoptive Cell Therapy in Cutaneous Melanoma: A Meta-Analysis. Oncologist.

[8] Verdon D.J., Jenkins M.R. (2021). Identification and Targeting of Mutant Peptide Neoantigens in Cancer Immunotherapy. Cancers.

[9] Hamieh M., Chatillon J.F., Dupel E., Bayeux F., Fauquembergue E., Maby P., Drouet A., Duval-Modeste A.B., Adriouch S., Boyer O. (2021). Generation of Pure Highly Functional Human Anti-Tumor Specific Cytotoxic T Lymphocytes with Stem Cell-Like Memory Features for Melanoma Immunotherapy. Front. Immunol..

[10] Bao M., Gempeler M., Campiche R. (2025). Melanosome Transport and Processing in Skin Pigmentation: Mechanisms and Targets for Pigmentation Modulation. Int. J. Mol. Sci..

[11] Li C., Kuai L., Cui R., Miao X. (2022). Melanogenesis and the Targeted Therapy of Melanoma. Biomolecules.

[12] Arrieta-Bolaños E., Hernández-Zaragoza D.I., Barquera R. (2023). An HLA map of the world: A comparison of HLA frequencies in 200 worldwide populations reveals diverse patterns for class I and class II. Front. Genet..

[13] Huuhtanen J., Chen L., Jokinen E., Kasanen H., Lönnberg T., Kreutzman A., Peltola K., Hernberg M., Wang C., Yee C. (2022). Evolution and modulation of antigen-specific T cell responses in melanoma patients. Nat. Commun..

[14] Benveniste P.M., Nakatsugawa M., Nguyen L., Ohashi P.S., Hirano N., Zuniga-Pflucker J.C. (2019). In vitro-generated MART-1-specific CD8 T cells display a broader T-cell receptor repertoire than ex vivo naïve and tumor-infiltrating lymphocytes. Immunol. Cell Biol..

[15] Pinto S., Sommermeyer D., Michel C., Wilde S., Schendel D., Uckert W., Blankenstein T., Kyewski B. (2014). Misinitiation of intrathymic MART-1 transcription and biased TCR usage explain the high frequency of MART-1-specific T cells. Eur. J. Immunol..

[16] Køllgaard T., Duval L., Schmidt H., Kaltoft K., Seremet T., Andersen M.H., Maase H.V.D., Thor Straten P., Hadrup S.R. (2009). Longitudinal immune monitoring of patients receiving intratumoral injection of a MART-1 T-cell receptor-transduced cell line (C-Cure 709). Cytotherapy.

[17] Biri-Kovács B., Bánóczi Z., Tummalapally A., Szabó I. (2023). Peptide Vaccines in Melanoma: Chemical Approaches towards Improved Immunotherapeutic Efficacy. Pharmaceutics.

[18] Borbulevych O.Y., Insaidoo F.K., Baxter T.K., Powell D.J., Johnson L.A., Restifo N.P., Baker B.M. (2007). Structures of MART-1(26/27-35) Peptide/HLA-A2 complexes reveal a remarkable disconnect between antigen structural homology and T cell recognition. J. Mol. Biol..

[19] Galloway S.A.E., Dolton G., Attaf M., Wall A., Fuller A., Rius C., Bianchi V., Theaker S., Lloyd A., Caillaud M.E. (2019). Peptide Super-Agonist Enhances T-Cell Responses to Melanoma. Front. Immunol..

[20] Madura F., Rizkallah P.J., Legut M., Holland C.J., Fuller A., Bulek A., Schauenburg A.J., Trimby A., Hopkins J.R., Wells S.A. (2019). TCR-induced alteration of primary MHC peptide anchor residue. Eur. J. Immunol..

[21] Du S., Yan J., Xue Y., Zhong Y., Dong Y. (2023). Adoptive cell therapy for cancer treatment. Exploration.

[22] Page M.J., McKenzie J.E., Bossuyt P.M., Boutron I., Hoffmann T.C., Mulrow C.D., Shamseer L., Tetzlaff J.M., Akl E.A., Brennan S.E. (2021). The PRISMA 2020 statement: An updated guideline for reporting systematic reviews. BMJ.

[23] Liu P., Zhou Z., Su X., Xu Y., Li J. (2026). Opportunities and challenges for cancer immunotherapy based on antigen cross-presentation. Ann. Med..

[24] Rohaan M.W., Gomez-Eerland R., van den Berg J.H., Geukes Foppen M.H., van Zon M., Raud B., Jedema I., Scheij S., de Boer R., Bakker N.A.M. (2022). MART-1 TCR gene-modified peripheral blood T cells for the treatment of metastatic melanoma: A phase I/IIa clinical trial. Immunooncol. Technol..

[25] Tsao H., Millman P., Linette G.P., Hodi F.S., Sober A.J., Goldberg M.A., Haluska F.G. (2002). Hypopigmentation associated with an adenovirus-mediated gp100/MART-1-transduced dendritic cell vaccine for metastatic melanoma. Arch. Dermatol..

[26] Block M.S., Nevala W.K., Pang Y.-P., Allred J.B., Strand C., Markovic S.N. (2019). A pilot clinical trial testing topical resiquimod and a xenopeptide as immune adjuvants for a melanoma vaccine targeting MART-1. Melanoma Res..

[27] Speiser D.E., Schwarz K., Baumgaertner P., Manolova V., Devevre E., Sterry W., Walden P., Zippelius A., Conzett K.B., Senti G. (2010). Memory and effector CD8 T-cell responses after nanoparticle vaccination of melanoma patients. J. Immunother..

[28] Tarhini A.A., Leng S., Moschos S.J., Yin Y., Sander C., Lin Y., Gooding W.E., Kirkwood J.M. (2012). Safety and immunogenicity of vaccination with MART-1 (26-35, 27L), gp100 (209-217, 210M), and tyrosinase (368-376, 370D) in adjuvant with PF-3512676 and GM-CSF in metastatic melanoma. J. Immunother..

[29] Jäger E., Höhn H., Necker A., Förster R., Karbach J., Freitag K., Neukirch C., Castelli C., Salter R.D., Knuth A. (2002). Peptide-specific CD8+ T-cell evolution in vivo: Response to peptide vaccination with Melan-A/MART-1. Int. J. Cancer.

[30] Jäger E., Ringhoffer M., Dienes H.P., Arand M., Karbach J., Jäger D., Ilsemann C., Hagedorn M., Oesch F., Knuth A. (1996). Granulocyte-macrophage-colony-stimulating factor enhances immune responses to melanoma-associated peptides in vivo. Int. J. Cancer.

[31] Adamina M., Rosenthal R., Weber W.P., Frey D.M., Viehl C.T., Bolli M., Huegli R.W., Jacob A.L., Heberer M., Oertli D. (2010). Intranodal immunization with a vaccinia virus encoding multiple antigenic epitopes and costimulatory molecules in metastatic melanoma. Mol. Ther..

[32] Rosenberg S.A., Zhai Y., Yang J.C., Schwartzentruber D.J., Hwu P., Marincola F.M., Topalian S.L., Restifo N.P., Seipp C.A., Einhorn J.H. (1998). Immunizing patients with metastatic melanoma using recombinant adenoviruses encoding MART-1 or gp100 melanoma antigens. J. Natl. Cancer Inst..

[33] Khammari A., Labarrière N., Vignard V., Nguyen J.-M., Pandolfino M.-C., Knol A.C., Quéreux G., Saïagh S., Brocard A., Jotereau F. (2009). Treatment of metastatic melanoma with autologous melan-a/mart-1-specific cytotoxic t lymphocyte clones. J. Investig. Dermatol..

[34] Vignard V., Lemercier B., Lim A., Pandolfino M.C., Guilloux Y., Khammari A., Rabu C., Echasserieau K., Lang F., Gougeon M.L. (2005). Adoptive transfer of tumor-reactive Melan-A-specific CTL clones in melanoma patients is followed by increased frequencies of additional Melan-A-specific T cells. J. Immunol..

[35] Dudley M.E., Wunderlich J.R., Robbins P.F., Yang J.C., Hwu P., Schwartzentruber D.J., Topalian S.L., Sherry R., Restifo N.P., Hubicki A.M. (2002). Cancer regression and autoimmunity in patients after clonal repopulation with antitumor lymphocytes. Science.

[36] Meidenbauer N., Marienhagen J., Laumer M., Vogl S., Heymann J., Andreesen R., Mackensen A. (2003). Survival and tumor localization of adoptively transferred Melan-A-specific T cells in melanoma patients. J. Immunol..

[37] Saberian C., Amaria R.N., Najjar A.M., Radvanyi L.G., Haymaker C.L., Forget M.A., Bassett R.L., Faria S.C., Glitza I.C., Alvarez E. (2021). Randomized phase II trial of lymphodepletion plus adoptive cell transfer of tumor-infiltrating lymphocytes, with or without dendritic cell vaccination, in patients with metastatic melanoma. J. Immunother. Cancer.

[38] Johnson L.A., Morgan R.A., Dudley M.E., Cassard L., Yang J.C., Hughes M.S., Kammula U.S., Royal R.E., Sherry R.M., Wunderlich J.R. (2009). Gene therapy with human and mouse T-cell receptors mediates cancer regression and targets normal tissues expressing cognate antigen. Blood.

[39] Seaman B.J., Guardiani E.A., Brewer C.C., Zalewski C.K., King K.A., Rudy S., Van Waes C., Morgan R.A., Dudley M.E., Yang J.C. (2012). Audiovestibular dysfunction associated with adoptive cell immunotherapy for melanoma. Otolaryngol. Head Neck Surg..

[40] van den Berg J.H., Gomez-Eerland R., van de Wiel B.A., Hulshoff L., van den Broek D., Bins A., Tan H.L., Harper J.V., Hassan N.J., Jakobsen B.K. (2015). Case Report of a Fatal Serious Adverse Event Upon Administration of T Cells Transduced with a MART-1-specific T-cell Receptor. Mol. Ther. J. Am. Soc. Gene Ther..

[41] Duval L., Schmidt H., Kaltoft K., Fode K., Jensen J.J., Sorensen S.M., Nishimura M.I., von der Maase H. (2006). Adoptive transfer of allogeneic cytotoxic T lymphocytes equipped with a HLA-A2 restricted MART-1 T-cell receptor: A phase I trial in metastatic melanoma. Clin. Cancer Res..

[42] Morgan R.A., Dudley M.E., Wunderlich J.R., Hughes M.S., Yang J.C., Sherry R.M., Royal R.E., Topalian S.L., Kammula U.S., Restifo N.P. (2006). Cancer regression in patients after transfer of genetically engineered lymphocytes. Science.

[43] Panelli M.C., Wunderlich J.R., Jeffries J., Wang E., Mixon A., Rosenberg S.A., Marincola F.M. (2000). Phase 1 Study in Patients With Metastatic Melanoma of Immunization With Dendritic Cells Presenting Epitopes Derived From the Melanoma-Associated Antigens MART-1 and gp100. J. Immunother..

[44] Butterfield L.H., Ribas A., Dissette V.B., Amarnani S.N., Vu H.T., Oseguera D., Wang H.J., Elashoff R.M., McBride W.H., Mukherji B. (2003). Determinant spreading associated with clinical response in dendritic cell-based immunotherapy for malignant melanoma. Clin. Cancer Res..

[45] Ribas A., Glaspy J.A., Lee Y., Dissette V.B., Seja E., Vu H.T., Tchekmedyian N.S., Oseguera D., Comin-Anduix B., Wargo J.A. (2004). Role of dendritic cell phenotype, determinant spreading, and negative costimulatory blockade in dendritic cell-based melanoma immunotherapy. J. Immunother..

[46] Palucka A.K., Ueno H., Connolly J., Kerneis-Norvell F., Blanck J.-P., Johnston D.A., Fay J., Banchereau J. (2006). Dendritic cells loaded with killed allogeneic melanoma cells can induce objective clinical responses and MART-1 specific CD8+ T-cell immunity. J. Immunother..

[47] Dannull J., Haley N.R., Archer G., Nair S., Boczkowski D., Harper M., De Rosa N., Pickett N., Mosca P.J., Burchette J. (2013). Melanoma immunotherapy using mature DCs expressing the constitutive proteasome. J. Clin. Investig..

[48] Geukes Foppen M.H., Rohaan M.W., Borgers J.S.W., Philips D., Vyth-Dreese F., Beijnen J.H., Nuijen B., van den Berg J.H., Haanen J.B.A.G. (2024). Intradermal Naked DNA Vaccination by DNA Tattooing for Mounting Tumor-Specific Immunity in Stage IV Melanoma Patients: A Phase I Clinical Trial. Oncol. Res. Treat..

[49] Triozzi P.L., Aldrich W., Allen K.O., Carlisle R.R., LoBuglio A.F., Conry R.M. (2005). Phase I study of a plasmid DNA vaccine encoding MART-1 in patients with resected melanoma at risk for relapse. J. Immunother..

[50] Weber J., Boswell W., Smith J., Hersh E., Snively J., Diaz M., Miles S., Liu X., Obrocea M., Qiu Z. (2008). Phase 1 trial of intranodal injection of a melan-A/MART-1 DNA plasmid vaccine in patients with stage IV melanoma. J. Immunother..

[51] Dréno B., Nguyen J.M., Khammari A., Pandolfino M.C., Tessier M.H., Bercegeay S., Cassidanius A., Lemarre P., Billaudel S., Labarrière N. (2002). Randomized trial of adoptive transfer of melanoma tumor-infiltrating lymphocytes as adjuvant therapy for stage III melanoma. Cancer Immunol. Immunother..

[52] Labarrière N., Pandolfino M.C., Gervois N., Khammari A., Tessier M.H., Dréno B., Jotereau F. (2002). Therapeutic efficacy of melanoma-reactive TIL injected in stage III melanoma patients. Cancer Immunol. Immunother..

[53] Benlalam H., Vignard V., Khammari A., Bonnin A., Godet Y., Pandolfino M.-C., Jotereau F., Dréno B., Labarrière N. (2007). Infusion of Melan-A/Mart-1 specific tumor-infiltrating lymphocytes enhanced relapse-free survival of melanoma patients. Cancer Immunol. Immunother..

[54] Yee C., Thompson J.A., Byrd D., Riddell S.R., Roche P., Celis E., Greenberg P.D. (2002). Adoptive T cell therapy using antigen-specific CD8+ T cell clones for the treatment of patients with metastatic melanoma: In vivo persistence, migration, and antitumor effect of transferred T cells. Proc. Natl. Acad. Sci. USA.

[55] Tüettenberg A., Becker C., Hüter E., Knop J., Enk A.H., Jonuleit H. (2006). Induction of strong and persistent MelanA/MART-1-specific immune responses by adjuvant dendritic cell-based vaccination of stage II melanoma patients. Int. J. Cancer.

[56] Terheyden P., Schrama D., Pedersen L.Ø., Andersen M.H., Kämpgen E., Thor Straten P., Becker J.C. (2003). Longitudinal Analysis of MART-1/HLA-A2-Reactive T Cells over the Course of Melanoma Progression. Scand. J. Immunol..

[57] Andersen M.H., Keikavoussi P., Bröcker E.B., Schuler-Thurner B., Jonassen M., Søndergaard I., Straten P.T., Becker J.C., Kämpgen E. (2001). Induction of systemic CTL responses in melanoma patients by dendritic cell vaccination: Cessation of CTL responses is associated with disease progression. Int. J. Cancer.

[58] Butterfield L.H., Comín-Anduix B., Vujanović L., Lee Y., Dissette V.B., Yang J.-Q., Vu H.T., Seja E., Oseguera D.K., Potter D.M. (2008). Adenovirus MART-1-engineered autologous dendritic cell vaccine for metastatic melanoma. J. Immunother..

[59] Duinkerken C.W., Rohaan M.W., de Weger V.A., Lohuis P.J.F.M., Latenstein M.N., Theunissen E.A.R., Balm A.J.M., Dreschler W.A., Haanen J.B.A.G., Zuur C.L. (2019). Sensorineural Hearing Loss after Adoptive Cell Immunotherapy for Melanoma Using MART-1 Specific T Cells: A Case Report and Its Pathophysiology. Otol. Neurotol..

[60] Reynolds S.R., Oratz R., Shapiro R.L., Hao P., Yun Z., Fotino M., Vukmanović S., Bystryn J.C. (1997). Stimulation of CD8+ T cell responses to MAGE-3 and Melan A/MART-1 by immunization to a polyvalent melanoma vaccine. Int. J. Cancer.

[61] Baumgaertner P., Costa Nunes C., Cachot A., Maby-El Hajjami H., Cagnon L., Braun M., Derré L., Rivals J.P., Rimoldi D., Gnjatic S. (2016). Vaccination of stage III/IV melanoma patients with long NY-ESO-1 peptide and CpG-B elicits robust CD8+ and CD4+ T-cell responses with multiple specificities including a novel DR7-restricted epitope. Oncoimmunology.

[62] Legat A., Maby-El Hajjami H., Baumgaertner P., Cagnon L., Abed Maillard S., Geldhof C., Iancu E.M., Lebon L., Guillaume P., Dojcinovic D. (2016). Vaccination with LAG-3Ig (IMP321) and Peptides Induces Specific CD4 and CD8 T-Cell Responses in Metastatic Melanoma Patients—Report of a Phase I/IIa Clinical Trial. Clin. Cancer Res..

[63] Ayyoub M., Zippelius A., Pittet M.J., Rimoldi D., Valmori D., Cerottini J.-C., Romero P., Lejeune F., Liénard D., Speiser D.E. (2003). Activation of human melanoma reactive CD8+ T cells by vaccination with an immunogenic peptide analog derived from Melan-A/melanoma antigen recognized by T cells-1. Clin. Cancer Res. Off. J. Am. Assoc. Cancer Res..

[64] Di Pucchio T., Pilla L., Capone I., Ferrantini M., Montefiore E., Urbani F., Patuzzo R., Pennacchioli E., Santinami M., Cova A. (2006). Immunization of stage IV melanoma patients with Melan-A/MART-1 and gplOO peptides plus IFN-α results in the activation of specific CD8+ T cells and monocyte/dendritic cell precursors. Cancer Res..

[65] Liénard D., Avril M.-F., Le Gal F.-A., Baumgaertner P., Vermeulen W., Blom A., Geldhof C., Rimoldi D., Pagliusi S., Romero P. (2009). Vaccination of melanoma patients with Melan-A/Mart-1 peptide and klebsiella outer membrane protein P40 as an adjuvant. J. Immunother..

[66] Urbani F., Ferraresi V., Capone I., Macchia I., Palermo B., Nuzzo C., Torsello A., Pezzotti P., Giannarelli D., Pozzi A.F. (2020). Clinical and Immunological Outcomes in High-Risk Resected Melanoma Patients Receiving Peptide-Based Vaccination and Interferon Alpha, With or Without Dacarbazine Preconditioning: A Phase II Study. Front. Oncol..

[67] Nisticò P., Capone I., Palermo B., Del Bello D., Ferraresi V., Moschella F., Aricò E., Valentini M., Bracci L., Cognetti F. (2009). Chemotherapy enhances vaccine-induced antitumor immunity in melanoma patients. Int. J. Cancer.

[68] Wang F., Bade E., Kuniyoshi C., Spears L., Jeffery G., Marty V., Groshen S., Weber J. (1999). Phase I trial of a MART-1 peptide vaccine with incomplete Freund’s adjuvant for resected high-risk melanoma. Clin. Cancer Res. Off. J. Am. Assoc. Cancer Res..

[69] Cormier J.N., Salgaller M.L., Prevette T., Barracchini K.C., Rivoltini L., Restifo N.P., Rosenberg S.A., Marincola F.M. (1997). Enhancement of cellular immunity in melanoma patients immunized with a peptide from MART-1/Melan A. Cancer J. Sci. Am..

[70] Goldinger S.M., Dummer R., Baumgaertner P., Mihic-Probst D., Schwarz K., Hammann-Haenni A., Willers J., Geldhof C., Prior J.O., Kündig T.M. (2012). Nano-particle vaccination combined with TLR-7 and -9 ligands triggers memory and effector CD8+ T-cell responses in melanoma patients. Eur. J. Immunol..

[71] Romano E., Michielin O., Voelter V., Laurent J., Bichat H., Stravodimou A., Romero P., Speiser D.E., Triebel F., Leyvraz S. (2014). MART-1 peptide vaccination plus IMP321 (LAG-3Ig fusion protein) in patients receiving autologous PBMCs after lymphodepletion: Results of a Phase I trial. J. Transl. Med..

[72] Ribas A., Weber J.S., Chmielowski B., Comin-Anduix B., Lu D., Douek M., Ragavendra N., Raman S., Seja E., Rosario D. (2011). Intra-lymph node prime-boost vaccination against Melan A and tyrosinase for the treatment of metastatic melanoma: Results of a phase 1 clinical trial. Clin. Cancer Res..

[73] Reeves E., James E. (2017). Antigen processing and immune regulation in the response to tumours. Immunology.

[74] Brunnberg J., Barends M., Frühschulz S., Winter C., Battin C., de Wet B., Cole D.K., Steinberger P., Tampé R. (2024). Dual role of the peptide-loading complex as proofreader and limiter of MHC-I presentation. Proc. Natl. Acad. Sci. USA.

[75] Lee M.Y., Jeon J.W., Sievers C., Allen C.T. (2020). Antigen processing and presentation in cancer immunotherapy. J. Immunother. Cancer.

[76] Sengupta D., Graham M., Liu X., Cresswell P. (2019). Proteasomal degradation within endocytic organelles mediates antigen cross-presentation. EMBO J..

[77] Buonaguro L., Tagliamonte M. (2023). Peptide-based vaccine for cancer therapies. Front. Immunol..

[78] Gu Y.Z., Zhao X., Song X.R. (2020). Ex vivo pulsed dendritic cell vaccination against cancer. Acta Pharmacol. Sin..

[79] Yee C., Thompson J.A., Roche P., Byrd D.R., Lee P.P., Piepkorn M., Kenyon K., Davis M.M., Riddell S.R., Greenberg P.D. (2000). Melanocyte destruction after antigen-specific immunotherapy of melanoma: Direct evidence of T cell-mediated vitiligo. J. Exp. Med..

[80] Fioretti D., Iurescia S., Fazio V.M., Rinaldi M. (2010). DNA vaccines: Developing new strategies against cancer. J. Biomed. Biotechnol..

[81] Morisaki T., Morisaki T., Kubo M., Morisaki S., Nakamura Y., Onishi H. (2022). Lymph Nodes as Anti-Tumor Immunotherapeutic Tools: Intranodal-Tumor-Specific Antigen-Pulsed Dendritic Cell Vaccine Immunotherapy. Cancers.

[82] Travieso T., Li J., Mahesh S., Mello J.D.F.R.E., Blasi M. (2022). The use of viral vectors in vaccine development. npj Vaccines.

[83] Liu W., Tang H., Li L., Wang X., Yu Z., Li J. (2021). Peptide-based therapeutic cancer vaccine: Current trends in clinical application. Cell Prolif..

[84] Grenier J.M., Yeung S.T., Qiu Z., Jellison E.R., Khanna K.M. (2018). Combining Adoptive Cell Therapy with Cytomegalovirus-Based Vaccine Is Protective against Solid Skin Tumors. Front. Immunol..

[85] Abakushina E.V., Popova L.I., Zamyatnin A.A., Werner J., Mikhailovsky N.V., Bazhin A.V. (2021). The Advantages and Challenges of Anticancer Dendritic Cell Vaccines and NK Cells in Adoptive Cell Immunotherapy. Vaccines.

[86] Alvarez-Dominguez C., Calderón-Gonzalez R., Terán-Navarro H., Salcines-Cuevas D., Garcia-Castaño A., Freire J., Gomez-Roman J., Rivera F. (2017). Dendritic cell therapy in melanoma. Ann. Transl. Med..

[87] Trakatelli M., Toungouz M., Lambermont M., Heenen M., Velu T., Bruyns C. (2005). Immune characterization of clinical grade-dendritic cells generated from cancer patients and genetically modified by an ALVAC vector carrying MAGE minigenes. Cancer Gene Ther..

[88] Zong J., Keskinov A.A., Shurin G.V., Shurin M.R. (2016). Tumor-derived factors modulating dendritic cell function. Cancer Immunol. Immunother..

[89] Xie Y.J., Liu W.Q., Li D., Hou J.C., Coghi P.S., Fan X.X. (2023). Overcoming Suppressive Tumor Microenvironment by Vaccines in Solid Tumor. Vaccines.

[90] Oliveira G., Stromhaug K., Klaeger S., Kula T., Frederick D.T., Le P.M., Forman J., Huang T., Li S., Zhang W. (2021). Phenotype, specificity and avidity of antitumour CD8+ T cells in melanoma. Nature.

[91] Tjin E.P.M., Konijnenberg D., Krebbers G., Mallo H., Drijfhout J.W., Franken K.L.M.C., van der Horst C.M.A.M., Bos J.D., Nieweg O.E., Kroon B.B.R. (2011). T-cell immune function in tumor, skin, and peripheral blood of advanced stage melanoma patients: Implications for immunotherapy. Clin. Cancer Res..

[92] Kazemi M.H., Sadri M., Najafi A., Rahimi A., Baghernejadan Z., Khorramdelazad H., Falak R. (2022). Tumor-infiltrating lymphocytes for treatment of solid tumors: It takes two to tango?. Front. Immunol..

[93] Karapetyan L. (2025). Infusion product characteristics to predict response to tumor-infiltrating lymphocyte (TIL) therapy in metastatic melanoma (MM). J. Clin. Oncol..

[94] Jiang Y., Li Y., Zhu B. (2015). T-cell exhaustion in the tumor microenvironment. Cell Death Dis..

[95] van Oijen M., Bins A., Elias S., Sein J., Weder P., de Gast G., Mallo H., Gallee M., Van Tinteren H., Schumacher T. (2004). On the role of melanoma-specific CD8+ T-cell immunity in disease progression of advanced-stage melanoma patients. Clin. Cancer Res..

[96] Yang J.C. (2015). Toxicities Associated With Adoptive T-Cell Transfer for Cancer. Cancer J..

[97] Zhao Y., Deng J., Rao S., Guo S., Shen J., Du F., Wu X., Chen Y., Li M., Chen M. (2022). Tumor Infiltrating Lymphocyte (TIL) Therapy for Solid Tumor Treatment: Progressions and Challenges. Cancers.

[98] Lin P., Lin Y., Mai Z., Zheng Y., Zheng J., Zhou Z., Zhao X., Cui L. (2025). Targeting cancer with precision: Strategical insights into TCR-engineered T cell therapies. Theranostics.

[99] Stone J.D., Harris D.T., Kranz D.M. (2015). TCR affinity for p/MHC formed by tumor antigens that are self-proteins: Impact on efficacy and toxicity. Curr. Opin. Immunol..

[100] Borbulevych O.Y., Santhanagopolan S.M., Hossain M., Baker B.M. (2011). TCRs used in cancer gene therapy cross-react with MART-1/Melan-A tumor antigens via distinct mechanisms. J. Immunol..

[101] Schmidt K., Leisegang M., Kloetzel P.M. (2023). ERAP2 supports TCR recognition of three immunotherapy targeted tumor epitopes. Mol. Immunol..

[102] Wang X., Song X., Li Y., Ding Y., Yin C., Ren T., Zhang W. (2025). Integrated system for screening tumor-specific TCRs, epitopes, and HLA subtypes using single-cell sequencing data. J. Immunother. Cancer.

[103] Madura F., Rizkallah P.J., Miles K.M., Holland C.J., Bulek A.M., Fuller A., Schauenburg A.J.A., Miles J.J., Liddy N., Sami M. (2013). T-cell receptor specificity maintained by altered thermodynamics. J. Biol. Chem..

[104] Jorritsma A., Gomez-Eerland R., Dokter M., van de Kasteele W., Zoet Y.M., Doxiadis I.I.N., Rufer N., Romero P., Morgan R.A., Schumacher T.N.M. (2007). Selecting highly affine and well-expressed TCRs for gene therapy of melanoma. Blood.

[105] Gomez-Eerland R., Nuijen B., Heemskerk B., van Rooij N., van den Berg J.H., Beijnen J.H., Uckert W., Kvistborg P., Schumacher T.N., Haanen J.B.A.G. (2014). Manufacture of gene-modified human T-cells with a memory stem/central memory phenotype. Hum. Gene Ther. Methods.

[106] Park M.Y., Kim C.H., Sohn H.J., Oh S.T., Kim S.G., Kim T.G. (2007). The optimal interval for dendritic cell vaccination following adoptive T cell transfer is important for boosting potent anti-tumor immunity. Vaccine.

[107] Song S., Zhang K., You H., Wang J., Wang Z., Yan C., Liu F. (2010). Significant anti-tumour activity of adoptively transferred T cells elicited by intratumoral dendritic cell vaccine injection through enhancing the ratio of CD8+ T cell/regulatory T cells in tumour. Clin. Exp. Immunol..

[108] Lou Y., Wang G., Lizée G., Kim G.J., Finkelstein S.E., Feng C., Restifo N.P., Hwu P. (2004). Dendritic cells strongly boost the antitumor activity of adoptively transferred T cells in vivo. Cancer Res..

[109] Chodon T., Comín-Anduix B., Chmielowski B., Koya R.C., Wu Z., Auerbach M., Ng C., Avramis E., Seja E., Villanueva A. (2014). Adoptive transfer of MART-1 T-cell receptor transgenic lymphocytes and dendritic cell vaccination in patients with metastatic melanoma. Clin. Cancer Res. Off. J. Am. Assoc. Cancer Res..

[110] Foley K.C., Nishimura M.I., Moore T.V. (2018). Combination immunotherapies implementing adoptive T-cell transfer for advanced-stage melanoma. Melanoma Res..

[111] Verdegaal E.M.E. (2016). Adoptive cell therapy: A highly successful individualized therapy for melanoma with great potential for other malignancies. Curr. Opin. Immunol..

