# Supplementary material for: Efficacy and Safety of Immunotherapeutic Strategies for the Treatment of MART-1-Expressing Melanoma: Systematic Review of Clinical Trials
Source: Cancers (Basel). 2026 Sep 17;18(18):3016. doi: 10.3390/cancers18183016 (PMC13605955; doi:10.3390/cancers18183016)
Supplement: Supplementary file 1 [file cancers-18-03016-s001.zip › cancers-4500247-Table S1.html]

## Table S1. Characteristics and outcomes of the clinical trials included in the review

*Each trial is represented by the first author of the corresponding publication and/or its registry identifier. The last search was performed on 15 January 2026.
In the accompanying circular diagram, the color varies according to the objective response rate,
calculated for patients with measurable disease evaluable by RECIST criteria.
The size of each circle corresponds to the number of patients treated in the trial.
Larger circles indicate larger sample sizes. The color scale for objective response rate:*

– 0%


– 0.1–10%


– 10–30%


– >30%
(NR – not reported)

| Trial | Therapy | Data source | Start year | Phase | Patients treated (n) | Patients evaluable for response (n) | Survival (median, days) | ORR (n/N) | Diagram |
| --- | --- | --- | --- | --- | --- | --- | --- | --- | --- |
| Non-adoptive cell immunotherapy | | | | | | | | | |
| Peptide vaccines | | | | | | | | | |
| NCT00651703  Goldinger et al. (PMID: 22806397) | MelQbG10 (VLP-like nanoparticlle loaded with CpG-ODN coupled to MART-1 16-35) + Montanide ISA-51 + Imiquimod. | Publication + registry record | 2008 | IIa | 21 | 14 | NR | 0% (0/14) |  |
| Di Pucchio et al. (PMID: 16651452) | MART-1: 26-35 (27L) + gp100: 209-217 (210M) + INF-alpha. | Publication | NS | I-II | 7 | 5 | NR | 0% (0/5) |  |
| NCT00059475 | Arm I: MART-1: 27-35 in Montanide ISA 51: Arm II: MART-1: 27-35 (27L) (Mod9mer) in Montanide ISA 51;  Arm III: MART-1: 26-35 (27L) (Mod10mer) in Montanide ISA 51;  Arm IV: MART-1: 27-35 (27L) + gp100: 209-217(210M) in Montanide ISA 51. | Registry record only | 2003 | II | 124 | NR | NR | NR |  |
| Baumgaertner et al. (PMID: 27853637)  NCT00112242 | Arm I: Melan-A ELA Montanide ISA-51; Arm II: Melan-A ELA + NY-ESO-1b(A) + MAGE-A10 Montanide ISA-51; Arm III: Melan-A ELA + NY-ESO-1b(A) + MAGE-A10 + Montanide ISA-51 + CpG; Arm IV: Melan-A EAA/ELA (if HLA-A2+) or NY-ESO-1lp + MAGE-A10 + Montanide ISA-51 + CpG; Arm V: Melan-A EAA/ELA (if HLA-A2+) or NY-ESO-1lp + MAGE-A10 + Montanide ISA-51+ CpG + IL-2.  *Contributes three cohorts to the epitope and antigen subgroups (n = 19, n = 10 and n = 10).* | Publication + registry record | 2004 | I | 39 | NR | NR | NR |  |
| Speiser et al. (PMID: 20842051)  3 substudies: NCT00306514 NCT00306566 NCT00306553 | MelQbG10 (MART-1 16-35 (A27L) + CpG-ODN) + Montanide ISA-51 | Publication + registry record | 2006 | I-II | 22 | 15 | NR | 6.7% (1/15) |  |
| NCT00515528 | Arm I: MART-1, gp100, MAGE-3, NA17, GM-CSF in Montanide; Arm II: MART-1, gp100, MAGE-3, NA17, GM-CSF in Montanide peptide vaccine + Ontak. | Registry record only | 2007 | II | 17 | 17 | NR | 5.9% (1/17) |  |
| Legat et al. (PMID: 26500235)  NCT01308294 | Vaccine injections (NY-ESO-1, MAGE-3.A2, NA-17 peptides + IMP321 + MART-1 + MAGE-A3-DP4 peptides + IMP321 + Montanide) in one limb or in distinct limbs. | Publication + registry record | 2010 | I-II | 16 | 4 | NR | 0% (0/4) |  |
| NCT00471471  Tarhini et al. (PMID: 22495394) | MART-1 (26–35, 27L), gp100 (209–217, 210M), tyrosinase (368–376, 370D), combined with the GM-CSF and PF3512676, emulsified in Montanide ISA. | Publication + registry record | 2008 | I | 21 | 21 | PFS: 56.9 OS: 407.9 | 9.5% (2/21) |  |
| Jäger et al. (PMID: 8690525, 11920589) | Peptide vaccine with MART-1-derived peptides, tyrosinase, and gp100. | Publication | 1995 | NS | 3 | 3 | NR | 100% (3/3) |  |
| Lienard et al. (PMID: 19752746) | MART-1 (ELA) + P40 deried from Klebsiella pneumoniae. | Publication | 2003 | I | 14 | 9 | NR | 0% (0/9) |  |
| Reynolds et al. (PMID: 9378560) | MAGE-3 and MART-1 peptides with either alum or IL-2 liposomes. | Publication | NS | NS | 15 | NR | RFS: 456.6 | NR |  |
| Wang et. al (PMID: 10537339) | MART-1 27–35 complexed with incomplete Freund's adjuvant. | Publication |  | I | 25 | NR | NR | NR |  |
| Cormier et al. (PMID: 9072306) | Arm I: MART-1 27–35 in incomplete Freund’s adjuvant. | Publication | NS | I | 23 | 23 | NR | 0% (0/23) |  |
| NCT01307618 | Arm I: peptide vaccine (NA17.A2, MAGE-3, MART-1 emulsified with Montanide ISA-51); Arm II: peptide vaccine (NA17.A2, MAGE-3 emulsified with Montanide ISA-51) + IL-12. | Registry record only | 2011 | II | 10 | NR | PFS: Arm I: 130, Arm II: 64 OS: Arm I: 804, Arm II: 199 | NR |  |
| NCT00084656 | Arm I: Ipilimumab + Tyrosinase/gp100/MART-1 emulsified in Montanide ISA 51 VG; Arm II: Ipilimumab + Tyrosinase/gp100/MART-1 emulsified in Montanide ISA 51 VG. | Registry record only | 2004 | II | 76 | NR | NR | NR |  |
| NCT01989572 | Arm I (GM-CSF; tyrosinase, gp100, MART-1 peptide vaccine in IFA /Montanide ISA-51 VG; Arm II (GM-CSF placebo; tyrosinase, gp100, MART-1 peptide vaccine emulsified in IFA /Montanide ISA-51 VG). | Registry record only | 2000 | III | 220 | NR | RFS: 349.6 OS: 2085.4 | NR |  |
| Nisticò et al. (PMID: 18839429) | Arm I: MART-1: 26–35 (A27L), gp100: 209–217 (210M) peptides in Montanide ISA-51 + IFN-alpha; Arm II: DTIC + MART-1: 26–35: 26–35 (A27L), gp100: 209–217 (210M) peptides in Montanide ISA-51 + IFN-alpha. | Publication | NS | I-II | 10 | NR | RFS: 212.8 OS: 881.6 | NR |  |
| Urbani et al. (PMID:32211314)  2008-008211-26 (EU Clinical Trials Register) | Arm I: MART-1 + NY-ESO-1 + IFN-α2b + dacarbazine; Arm II: MART-1 + NY-ESO-1 + IFN-α2b. | Publication + registry record | 2009 | II | 34 | NR | RFS: 347 OS: not reached (f.u.: 4.5 years) | NR |  |
| Ayyoub et al. (PMID: 12576434) | Arm I: MART-1 26–35 (ELA) + Influenza matrix peptide 58–66 + QS21-based immune adjuvant AS02B. | Publication | NS | I | 3 | NR | NR | NR |  |
| DNA vaccines | | | | | | | | | |
| Adamina et al. (PMID: 19935776)  NCT00116597 | Recombinant vaccinia virus encoding gp100 280–288, MART-1 27–35, tyrosinase 1–9, and CD80/CD86 + GM-CSF. | Publication + registry record | 2002 | I-II | 15 | 4 | PFS: 310 OS: 488.5 | 25% (1/4) |  |
| Triozzi et al. (PMID: 16000957) | Arm I: Plasmid DNA construct encoding MART-l + plasmid encoding hepatitis B surface antigen intramusculary. | Publication | NS | I | 12 | NR | NR | NR |  |
| Rosenberg et al. (PMID: 9862627) | Recombinant adenovirus encoding MART-1 with or w/o IL-2. | Publication | NS | I | 36 | 36 | NR | 13.9% (5/36) |  |
| Geukes Foppen et al. (PMID: 38583422) | DNA vaccine encoding MART-1 26–35 (ELA) and tetanus toxin fragment C (pDERMATT). | Publication | 2009 | I | 9 | 9 | PFS: 54.79 OS: 179.6 | 0% (0/9) |  |
| NCT00706992 | Arm VII: ALVAC MART-1 vaccine. | Registry record only | 2008 | II | 6 | NR | NR | NR |  |
| Weber et al. (PMID: 18481391) | Vaccine containing pSEM plasmide encoding MART-1 26-35 (ELA) and 31-70 and Tyrosinase 1-9 and 369-377 epitopes. | Publication | NS | I | 19 | NR | TTP: 61.5 OS: 334.84 | NR |  |
| Adoptive cell immunotherapy | | | | | | | | | |
| Autologous cell transfer | | | | | | | | | |
| NCT00001832 | Phase I: Cyclophosphamide, Fludarabine + Cells intravenous with or w/o IL-2; Phase II: Cyclophosphamide, Fludarabine + Cells intravenous or intraarterial with or w/o IL-2 + with or w/o G-CSF. | Registry record only | 1999 | II | 89 | 89 | NR | 25.8% (23/89) |  |
| NCT01495572 | Cyclophosphamide, Fludarabine + MART-1 27-35 reactive CD8+ PBL. | Registry record only | 2011 | II | 5 | 5 | NR | 0% (0/5) |  |
| NCT00720031  Khammari et al. (PMID: 19554023) | Dacarbazine + autologous MART-1 A27L-reactive PBMCs + INF-alpha + IL-2. | Publication + registry record | 2000 | I-II | 14 | 14 | NR | 42.9% (6/14) |  |
| Vignard et al. (PMID: 16177129) | Tumor-reactive MART-specific T cell clones + IL-2 + INF-alpha. | Publication | NS | I-II | 10 | 10 | NR | 10% (1/10) |  |
| Yee et al. (PMID: 12427970) | CD8+ T cell clones targeting MART1 and gp100 + IL-2. | Publication | NS | I | 5 | 5 | NR | 0% (0/5) |  |
| Dudley et al. (PMID: 12242449) | Cyclophosphamide and fludarabine + tumor-reactive T cells + IL-2. | Publication | NS | NS | 13 | 13 | NR | 46.2% (6/13) |  |
| Dréno et al. (PMID: 12384805)  Labarrière et al. (PMID: 12384804)  Benlalam et al. (PMID: 16874485) | TIL expanded from melanoma-invaded lymph nodes + IL-2. | Publication | 1994 | II-III | 44 | NR | RFS: 301.36 OS: 898.89 | NR |  |
| Meidenbauer et al. (PMID: 12574389) | MART-1-specific CD8+ T cells + IL-2. | Publication | NS | NS | 8 | 8 | NR | 12.5% (1/8) |  |
| Saberian et al. (PMID: 34021033)  NCT00338377 | MART-1 reactive CD8+ TIL. | Publication + registry record | 2006 | II | 10 | 10 | OS: 1496.5 PFS: 94.9 | 30% (3/10) |  |
| TCR-T therapy | | | | | | | | | |
| NCT00706992 | Arm I: F5 T-cells; Arm III: F5 T-cells + IL-2. | Registry record only | 2008 | II | 17 | NR | NR | NR |  |
| NCT00509288  Johnson et al. (PMID: 19451549)  Seaman et al. (PMID: 22597578) | Cyclophosphamide + Fludarabine + Anti-MART-1 F5 TCR transduced TILs/PBLs + IL-2. | Publication + registry record | 2007 | II | 20 | 20 | NR | 30% (6/20) |  |
| NCT02654821  Rohaan et al. (PMID: 35865122)  van den Berg et al. (PMID: 25896248) | Cyclophosphamide + fludarabine + IL-2 + 1D3HMCys TCR transduced PBLs. | Publication + registry record | 2008 | I-II | 11 | 11 | PFS: 85 OS: 222 | 18.2% (2/11) |  |
| Køllgaard et al. (PMID: 19530030)  Duval et al. (PMID: 16489078) | C-Cure 709 cells administered intratumorally. | Publication | 2002 | I | 15 | 15 | NR | 6.7% (1/15) |  |
| Duinkerken et al. (PMID: 31295198)  EudraCT no. 2011-002941-36 | Cyclophosphamide + fludarabine + TCR-modified T cells. | Case report | Not stated | I-II | 1 | 1 | NR | 0% (0/1) |  |
| Morgan et al. (PMID: 16946036) | Lymphodepletion + PBLs transduced with anti-MART-1 TCR. | Publication | NS | NS | 17 | 17 | NR | 11.8% (2/17) |  |
| Dendritic cell vaccines | | | | | | | | | |
| Tuettenberg et al. (PMID: 16353138) | DCs pulsed with MART-1 peptide and tetanus toxoid/tuberculin. | Publication | 2001 | NS | 13 | NR | NR | NR |  |
| Panelli et al. (PMID: 10916759) | DCs pulsed with MART-1(27-35), DCs pulsed with gp-100-209-2M with or w/o IL-2. | Publication | NS | I | 7 | 7 | NR | 14.3% (1/7) |  |
| Butterfield et al. (PMID: 12631598) | MART-1 27–35 peptide-pulsed autologous DCs intradermally or intravenously. | Publication | NS | I | 18 | 10 | NR | 10% (1/10) |  |
| Ribas et al. (PMID: 15314544) | MART-1 27-35 peptide-pulsed DCs intradermally. | Publication | NS | II | 9 | 4 | NR | 25% (1/4) |  |
| Butterfield et al. (PMID: 18317358) | AdVMART1-transduced DC vaccine with the MART-1 gene encoding full-lenght antigen. | Publication | NS | I-II | 18 | 17 | PFS: 182.64 OS: 365.28 | 0% (0/17) |  |
| Palucka et al. (PMID: 16971810) | Autologous monocyte-derived DCs loaded with killed allogeneic Colo829 melanoma cell line. | Publication | 2002 | I | 20 | 20 | OS: 684 | 10% (2/20) |  |
| Terheyden et al. (PMID: 14629628) | DCs pulsed with tumor peptide antigens from gp100, MART-1 and MAGE-3 alone or in combination with autologous oncolysates. | Case report | 1998 | NS | 1 | NR | NR | NR |  |
| Andersen et al. (PMID: 11745484) | Monocyte-derived dendritic cells pulsed with either antigens from gp100, MART-1, and MAGE-3 alone or in combination with autologous oncolysates. | Publication | NS | NS | 2 | 2 | NR | 0% (0/2) |  |
| Tsao et al. (PMID: 12056962) | DCs transduced with replication-deficient adenovirus encoding MART-1 and gp100. | Publication | NS | I | 12 | NR | NR | NR |  |
| NCT00672542   Dannull et al. (PMID: 23934126) | MART1, MAGE-3, gp100, and tyrosinase RNA–transfected DCs derived from iP siRNA–transfected monocytes. | Publication + registry record | 2008 | I | 12 | 2 | NR | 100% (2/2) |  |
| Combination (ACT+ACT) | | | | | | | | | |
| Chodon et al. (PMID: 24634374)  NCT00910650 | Cyclophosphamide + Fludarabine + F5 TCR transgenic cells + MART-1 26-35 loaded DCs. | Publication + registry record | 2009 | II | 13 | 13 | NR | 0% (0/13) |  |
| Saberian et al. (PMID: 34021033)  NCT00338377 | MART-1 reactive CD8+ TIL + DCs pulsed with MART-1 peptide MART-1 26-35 (27L) + IL-2. | Publication + registry record | 2006 | II | 8 | 8 | PFS: 178.85 OS: 730 | 50% (4/8) |  |
| Combination (ACT+non-ACT) | | | | | | | | | |
| NCT00923195 | Arm I: Fludarabine, Cyclophosphamide + Anti-gp 100:154 TCR PBL and anti-MART-1 TCR PBL + IL-2 + gp100:154-162 Peptide vaccine in Montanide ISA 51 VG; Arm II: Fludarabine, Cyclophosphamide + Anti-gp 100:154 TCR PBL and anti-MART-1 TCR PBL + IL-2 + MART-1:26-35(27L) Peptide vaccine in Montanide ISA 51 VG; Amendment D: Arm III: Fludarabine, Cyclophosphamide + Anti-gp 100:154 TCR PBL and anti-MART-1 TCR CD8+ PBL + IL-2 + gp100:154-162 Peptide vaccine in Montanide ISA 51 VG; Arm IV: Fludarabine, Cyclophosphamide + Anti-gp 100:154 TCR PBL and anti-MART-1 TCR CD8+ PBL + IL-2 + MART-1:26-35(27L) Peptide vaccine in Montanide ISA 51 VG. | Registry record only | 2008 | II | 4 | 4 | NR | 0% (0/4) |  |
| NCT00612222 | Cyclophosphamide + Fludarabine + ALVAC vaccine + IL-2 + anti-MART-1 F5 cells. | Registry record only | 2008 | II | 4 | 4 | NR | 0% (0/4) |  |
| NCT00706992 | Arm II: F5 T-cells + MART-1:26-35(27L) peptide in Montanide ISA-51 VG; Arm IV: F5 T-cells + MART-1:26-35(27L) peptide in Montanide ISA-51 VG + IL-2. | Registry record only | 2008 | II | 16 | NR | NR | NR |  |
| NCT00706992 | Arm V: F5 T-cells + ALVAC MART-1 vaccine; Arm VI: F5 T-cells + ALVAC vaccine + IL-2. | Registry record only | 2008 | II | 11 | NR | NR | NR |  |
| NCT00001832 | Cyclophosphamide + fludarabine + IL-2 + GMSF + gp100:209-217(210M) (in gp100-reactive patients) + MART-1:26-35(27L) (in MART-1-reactive patients). | Registry record only | 1999 | II | 18 | 18 | NR | 27.8% (5/18) |  |
| NCT00324623  Romano et al. (PMID: 24726012) | Arm I: Cyclophosphamide, Fludarabine + PBMC + MART-1 26–35 (A27L) in IFA-51; Arm II: Cyclophosphamide, Fludarabine + PBMC + MART-1 26–35 (A27L) in IFA-51 + IMP321. | Publication + registry record | 2005 | I | 12 | 12 | NR | 0% (0/12) |  |
| Combination (non-ACT+non-ACT) | | | | | | | | | |
| NCT00688090  Ribas et al. (PMID: 21385924) | MKC1106-MT regimen: plasmid pMEL-TYR encoding MART-1 26–35 A27L, 31–96 and tyrosinase 369–377; peptides: Melan A26–35 E-MEL (ENvaAGIGILTV)  tyrosinase 369–377 E-TYR (YMDGTMSQNva) | Publication + registry record | 2008 | I | 18 | 18 | NR | 0% (0/18) |  |

**Notes.**

1. The table contains 59 rows and 54 unique clinical trials. Trials that evaluated
different modalities in separate cohorts appear once per cohort, in the corresponding
modality block: NCT00706992 contributes four cohorts (DNA vaccine; TCR-T therapy;
TCR-T therapy with peptide vaccine; TCR-T therapy with DNA vaccine), NCT00001832 and
NCT00338377 contribute two cohorts each. The column "Patients treated" is therefore
cohort-level, and its sum over all rows is 1292, which is the total number of patients
in the review.

2. "Patients evaluable for response" are the patients of a given cohort who entered
the trial with measurable disease assessable by RECIST. Only these patients contribute
to the denominator of the objective response rate. Their sum over all rows is 517.
Cohorts for which no response data were reported are marked NR and contribute to the
patient count only.

3. "Data source" indicates whether the outcome data were taken from a peer-reviewed
publication, from a trial registry record for which no corresponding full-text
publication could be identified, or from a single-patient case report. Ten of the
54 trials were available only as registry records and two records are case reports;
this is the reason why a formal risk-of-bias assessment was not undertaken
(see Methods, Section 2.5).

4. Survival values are medians in days, extracted as reported or, where only
individual patient data were available, estimated by the Kaplan-Meier method.

ORR - objective response rate; NR - not reported; NS - not stated; PFS - progression-free
survival; OS - overall survival; TTP - time to progression; RFS - recurrence-free survival.
